# Supplementary material for: Examination of CIs in health and medical journals from 1976 to 2019: an observational study
Source: BMJ Open. 2019 Nov 21;9(11):e032506. doi: 10.1136/bmjopen-2019-032506 (PMC6887056; doi:10.1136/bmjopen-2019-032506)
Supplement: Supplementary data [file bmjopen-2019-032506supp001.pdf]

# Confidence intervals in health and medical journals show an implausible excess of statistically significant results

Adrian Barnett and Jonathan Wren

## S1. Histograms

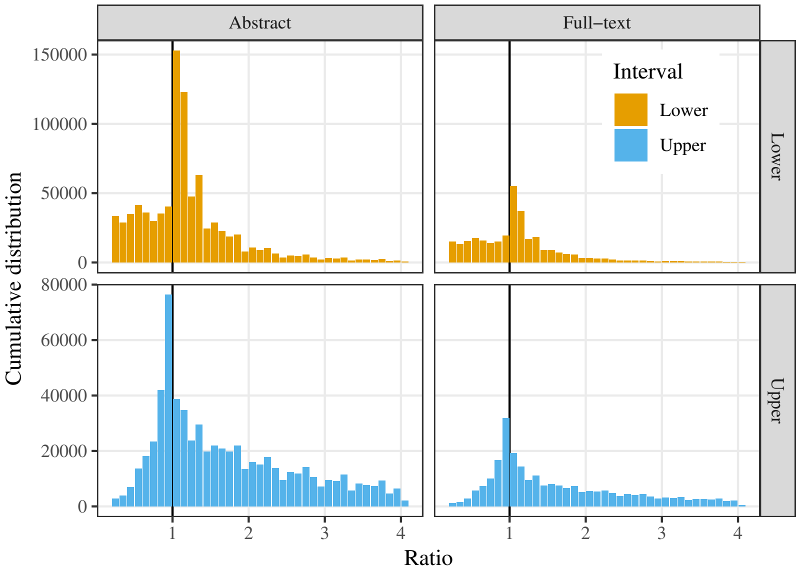


Figure S.1: Histograms for ratio confidence intervals from *Medline* abstracts and full-texts. The ratios are limited to 0.2 to 4.0.

The results in the figure above are an alternative version of the main results using a histogram rather than a cumulative density plot. We used binwidths of 0.1. The plot shows the dramatic rise in intervals just above 1 for the lower intervals, and rise in intervals below 1 for the upper intervals. The downside to using histograms is the requirement to select the binwidth and the plot's appearance does depend on this choice.



Barnett AG, Wren JD. *BMJ Open* 2019; 9:e032506. doi: 10.1136/bmjopen-2019-032506

## S2. Plots using a log-scale

In the results we focused on the key intervals close to 1 by restricting the intervals to between 0.25 and 4, which had the consequence of excluding 11% of lower intervals and 33% of upper intervals. The plot below uses a log-scale (base e) for the intervals which allows us to show ratios from 0.1 to 10. This plot excludes 4% of lower intervals and 15% of upper intervals.

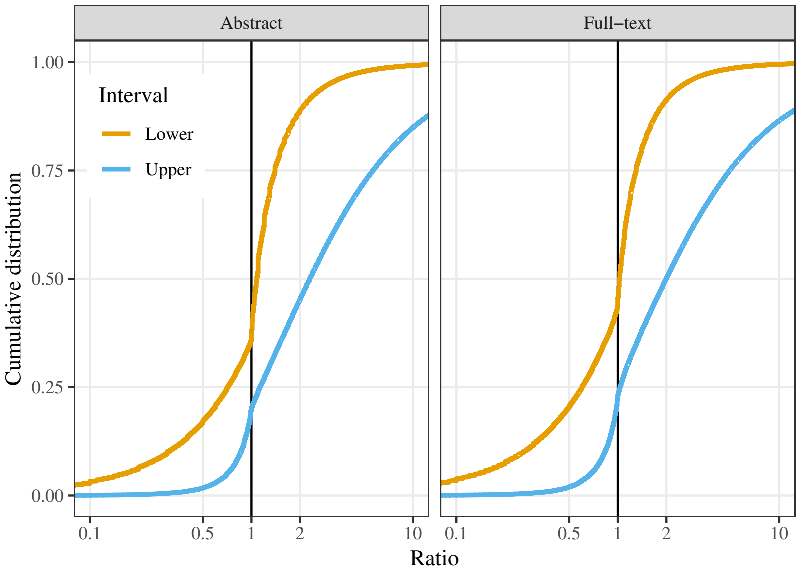


Figure S.2: Empirical cumulative distributions for ratio confidence intervals from *Medline* abstracts and full-texts. To be statistically significant, lower intervals need to be above 1 and upper intervals need to be under 1 (vertical lines). The x-axes are on a log-scale (base e).

The figure shows the same dramatic change close to 1 for the lower and upper intervals. One advantage of showing more of the data is that it confirms that the only discontinuity is at a ratio of 1.



Barnett AG, Wren JD. *BMJ Open* 2019; 9:e032506. doi: 10.1136/bmjopen-2019-032506

## S3. Intervals in a narrow range close to 1

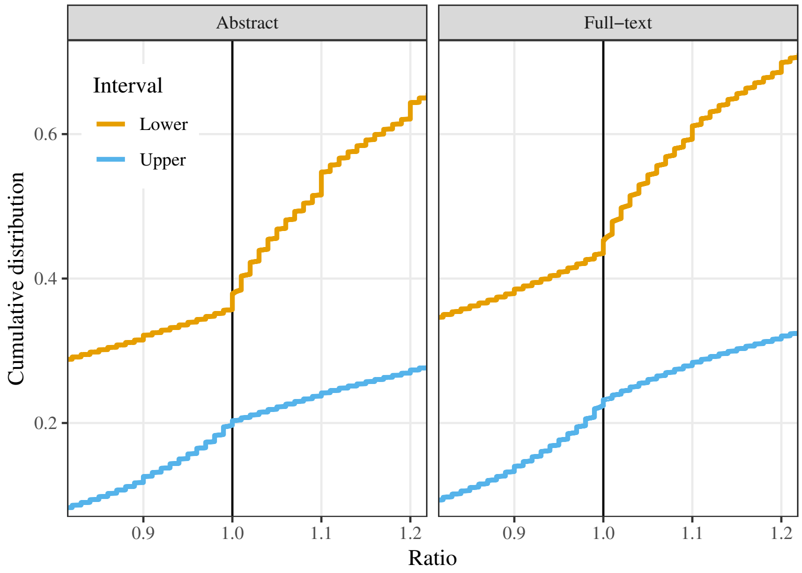


Figure S.3: Empirical cumulative distributions for ratio confidence intervals from *Medline* abstracts and full-texts. The ratios are limited to the narrow range of 0.83 to 1.2. The y-axes do not start at zero.

The plot above is an alternative look at the main results which examines those intervals close to 1. The x-axes are restricted to 0.83 to 1.2 and the y-axis are restricted to reduce white space. The plot includes 35% of all lower intervals and 20% of all upper intervals. The steps in the ratios are due to results being presented to 1 decimal place. The discontinuity in the cumulative numbers at 1 is still clearly visible and hence the changes shown in previous figures are not simply due to rounding.



Barnett AG, Wren JD. *BMJ Open* 2019; 9:e032506. doi: 10.1136/bmjopen-2019-032506

## S4. Full-text results over time

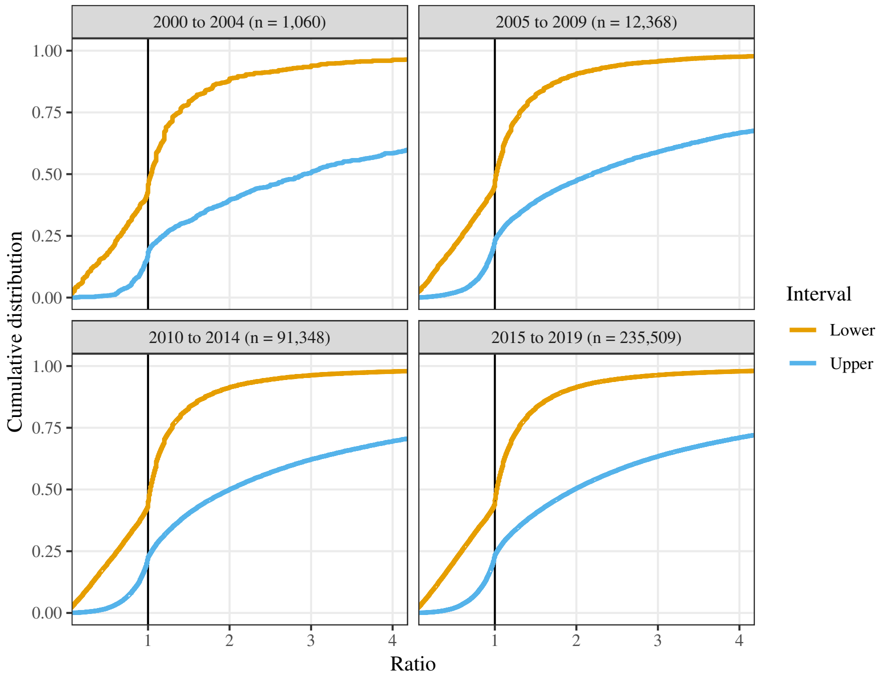


Figure S.4: Empirical cumulative distributions for ratio confidence intervals from full-texts in five year intervals. To be statistically significant, lower intervals need to be above 1 and upper intervals need to be under 1 (vertical lines).

The figure above is the complement to Figure 3 in the main paper, which examined the results over time from abstracts. This plot has a narrower time range because reasonable numbers of confidence intervals could only be extracted from the year 2000 onward. There was little change over time in the cumulative distributions, indicating little improvement in the bias for statistically significant results.



Barnett AG, Wren JD. *BMJ Open* 2019; 9:e032506. doi: 10.1136/bmjopen-2019-032506
