# Supplementary material for: Examination of CIs in health and medical journals from 1976 to 2019: an observational study
Source: BMJ Open. 2019 Nov 21;9(11):e032506. doi: 10.1136/bmjopen-2019-032506 (PMC6887056; doi:10.1136/bmjopen-2019-032506)
Supplement: Author's manuscript [file bmjopen-2019-032506.draft_revisions.pdf]

BMJ Open is committed to open peer review. As part of this commitment we make the peer review history of every article we publish publicly available.

When an article is published we post the peer reviewers' comments and the authors' responses online. We also post the versions of the paper that were used during peer review. These are the versions that the peer review comments apply to.

The versions of the paper that follow are the versions that were submitted during the peer review process. They are not the versions of record or the final published versions. They should not be cited or distributed as the published version of this manuscript.

BMJ Open is an open access journal and the full, final, typeset and author-corrected version of record of the manuscript is available on our site with no access controls, subscription charges or pay-per-view fees (http://bmjopen.bmj.com).

If you have any questions on BMJ Open's open peer review process please email info.bmjopen@bmj.com

BMJ Open

Confidence intervals in health and medical journals show an implausible excess of statistically significant results: an observational study

Journal:	*BMJ Open*
Manuscript ID	bmjopen-2019-032506
Article Type:	Research
Date Submitted by the Author:	21-Jun-2019
Complete List of Authors:	Barnett, Adrian; Queensland University of Technology, Institute of Health and Biomedical Innovation<br>Wren, Jonathan; Oklahoma Medical Research Foundation, Arthritis and Clinical Immunology Research Program, Division of Genomics and Data Sciences; University of Oklahoma Health Sciences Center, Department of Biochemistry and Molecular Biology
Keywords:	p-values, statistical significance, confidence intervals, p-hacking, publication bias

SCHOLARONE™
Manuscripts

Title page

Title: Confidence intervals in health and medical journals show an implausible excess of statistically significant results: an observational study

Adrian G. Barnett [1]

Jonathan D. Wren, [2,3]

[1] Institute of Health and Biomedical Innovation & School of Public Health and Social Work Queensland University of Technology, Australia

[2] Arthritis and Clinical Immunology Research Program, Division of Genomics and Data Sciences, Oklahoma Medical Research Foundation, Oklahoma City, OK, USA

[3] Department of Biochemistry and Molecular Biology, University of Oklahoma Health Sciences Center, Oklahoma City, OK, USA

Corresponding author's contact information:

Institute of Health and Biomedical Innovation & School of Public Health and Social Work

Queensland University of Technology

60 Musk Avenue

Kelvin Grove, Queensland, 4059

Australia

E-mail: a.barnett@qut.edu.au

Phone: ++61-7-3138 6010, Fax: ++61-7-3138 6030

Word count: 1661

For peer review only

Abstract

Objectives Previous research has shown clear biases in the distribution of published p-values, with an excess below the 0.05 threshold due to a combination of p-hacking and publication bias. We aimed to examine the bias for statistical significance using published confidence intervals.

Design Observational study.

Setting Papers published in *Medline* since 1976.

Participants Over 968,000 confidence intervals extracted from abstracts and over 350,000 intervals extracted from the full-text.

Outcome measures Cumulative distributions of lower and upper confidence interval limits for ratio estimates.

Results We found an excess of statistically significant results with a glut of lower intervals just above 1 and upper intervals just below 1. These excesses have not improved in recent years. The excesses did not appear in a set of over 100,000 confidence intervals that were not subject to p-hacking or publication bias.

Conclusions The huge excesses of published confidence intervals that are just below the statistically significant threshold are not statistically plausible. Large improvements in research practice are needed to provide more results that better reflect the truth.

Keywords p-values, statistical significance, confidence intervals, p-hacking, publication bias

Strengths and limitations of this study:

- This is the first study to examine the bias towards statistical significance using confidence intervals instead of p-values.
- We used a very large sample of confidence intervals from both abstracts and full-texts.
- We used a simple graphical summary and did not develop a new test to detect p-hacking or examine differences in papers around the statistically significant threshold.
- We have taken a broad look at the combined evidence and have not examined individual studies to show that they were biased.

Introduction

Every health and medical researcher is aware of the importance of statistically significant results, generally meaning a p-value less than 0.05. A study with statistically significant

For peer review only - http://bmjopen.bmj.com/site/about/guidelines.xhtml

results is easier to publish in a journal [1] and is often perceived as having more validity and importance [2]. This can lead some researchers to strive for statistically significant results by re-analysing data to get a p-value under 0.05, known as "p-hacking" [3, 4]. This striving for statistical significance is not always overt and can occur due to researchers making seemingly sensible scientific decisions [5]. It can also motivate researchers to carefully vet which hypotheses they pursue, selecting those they deem likely to yield significant results. For example, around 75% of scientific effort goes towards the 10% of genes already best characterized, suggesting widespread risk-aversion [6].

Journals are also somewhat responsible for the bias towards statistically significant results. Journals depend on readership, and journal editors know that readers tend to be more interested in striking results.

The combined "significance seeking" behaviour of researchers and journals has created a statistically implausible excess of published p-values just below the 0.05 threshold [1, 7, 8]. This warped evidence undermines the purpose of evidence-based practice, as "negative" studies too often go unpublished and some published effect estimates are too large because p-hacking inflates effect sizes (by conditioning on statistically significant results). This biased evidence can harm the public's health when policy decisions are made using incomplete or overly-optimistic evidence.

Examining published p-values using the "p-curve" distribution can be used to indicate that p-hacking has occurred [3]. In this paper we show that the same excess occurs in confidence intervals, which are a recommended alternative to p-values for presenting results, and have been advocated as a way to avoid "bright-line" thinking at the 0.05 p-value threshold [9, 10, 11]. We therefore take an alternative look at the already illustrated bias for statistical significance. However, we think this is worthwhile replication because this bias is a crucial problem for evidence-based medicine. Also using confidence intervals may lead to a wider understanding of the bias because researchers often misunderstand p-values [12]. We also show that the bias has not abated in recent years.

As a reminder, a p-value is the probability, under a specified model, of observing a test statistic as or more extreme than that observed in the data. A 95% confidence interval is a range that should contain the true value on 95% of occasions if the data generating process could be repeated many times. Most confidence intervals are given as 95% intervals, which correspond to a 0.05 p-value threshold.

For peer review only - http://bmjopen.bmj.com/site/about/guidelines.xhtml

Methods

We extracted ratio confidence intervals from the abstracts and full-texts of journals using regular expressions, for full details see Georgescu and Wren [8]. The text-mining algorithm was designed to recognise the typical ways in which ratio estimates (e.g., odds, hazard and risk ratios) and confidence intervals are presented. The results were independently validated using a separate text-mining algorithm developed by the first author (AGB). Random checks were made on approximately 5,000 abstracts and 500 full texts, as well as other checks on unusual results, e.g., extremely wide confidence intervals. The complete codes and data are available at https://github.com/jdwren/ASEC and https://github.com/agbarnett/intervals.

We excluded the 0.1% of lower intervals that were zero, assuming this was an error by the authors. We excluded the 0.7% of abstract intervals and 0.9% of full-text intervals where the mean was not within the confidence interval, as this meant either the interval or mean (or both) was incorrect.

We used a second set of published estimates from health research based on thousands of analyses with no p-hacking or publication bias [13]. Using four large databases of insurance claims, the study examined all possible pairs of 17 treatments for depression using 22 outcomes. Risks were estimated using survival analysis, giving hazard ratios. Treatment–Outcome pairs were categorised as “negative” where there was no evidence in the literature of any association. “Positive” associations were created by simulating an increased risk with a hazard ratio of 1.5. Details are in the paper by Schuemie et al [13], the key point for our analysis is that we have a large sample of ratio confidence intervals from Treatment–Outcome pairs that are not subject to any “significance seeking” by researchers or journals.

Statistical methods

To summarise the intervals we plotted the cumulative empirical distribution of the lower and upper confidence interval limits. We used the cumulative distribution because it highlights a change in the distribution, which is our key interest, and it does not require any user-defined tuning parameters. Histograms require the selection of a binwidth and density plots require user-defined smoothing parameters, which are subjective and will create different impressions depending on the choice made. We include histograms in the online supplement using a binwidth of 0.1, but our results focus on the cumulative distributions.

Our hypothesis was that there would be a large change in the intervals near 1, which is the

For peer review only - http://bmjopen.bmj.com/site/about/guidelines.xhtml

commonly used null hypothesis of no difference on a ratio scale. A lower confidence interval limit for a ratio estimate that is greater than 1 would mean a statistically significant result, as would an upper interval limit below 1. To look for changes over time, we plotted separate cumulative densities in five year periods.

We used R version 3.6.0 for all analyses [14].

Patient and Public Involvement

No patients or members of the public were involved in this study.

Results

Our sample had over 968,000 intervals extracted from *Medline* abstracts from over 5,900 journals, and over 350,000 intervals extracted from the full-text from over 2,700 journals. For 11% of intervals the level of the confidence interval could not be determined, most often because it was not given. Where an interval could be determined, 99.7% were 95% confidence intervals, which corresponds to the commonly used p-value threshold of 0.05.

We found a clear and sudden change in the cumulative distribution of both lower and upper interval limits at the statistically significant threshold of 1 (Figure 1). There was a steep increase in the number of lower interval limits just above 1, meaning they were just above the threshold for statistical significance. Similarly there was a steep increase in the number of upper interval limits just below 1, and so just inside the statistically significant threshold. The discontinuity at 1 appears slightly stronger (less smooth) in the abstract than the full-text and there are noticeably more lower intervals below 1 in the full-text, which suggests that the biases are stronger in the abstract than the full-text.

If there was no p-hacking and publication bias, the cumulative distributions would be smooth S-shapes with no discontinuities, and this is confirmed in Figure 2 using the data without these biases.

The bias for statistically significant results in abstracts has persisted over time as shown by the five-year plots in Figure 3. The plots also show the reduced use of rounded intervals over time, as the cumulative densities are smoother in later years.

The online supplement contain sensitivity analyses that: use wider and narrow ratio axes; show histograms instead of cumulative densities; and show plots of the cumulative densities over time for the full-text. All the plots show the same striking changes in the number of intervals near 1.

For peer review only

For peer review only - http://bmjopen.bmj.com/site/about/guidelines.xhtml

Discussion

The strong bias for statistically significant results is clearly visible in the confidence intervals of a large sample of abstracts and full-texts from health and medical journals and has persisted for decades. This bias undermines the evidence base and impairs good decision making by giving a false picture of the effectiveness of treatments and procedures. Such biased evidence can harm patients and the public when it gets translated into policy and practice.

Confidence intervals have been promoted as a way of avoiding the simplistic bright-line thinking that often accompanies p-values. However, our results show that even though people are frequently reporting confidence intervals, many are still focusing on statistical significance. A push towards the greater use of confidence intervals in journals in place of p-values may therefore not solve the ongoing problem of bright-line thinking [15].

Large improvements in research practice are needed to improve the quality of evidence. Study results should be presented and published regardless of their statistical significance. This could be achieved by a wider uptake of study pre-registration and analysis plans [16], which reduces the potential for researchers to change their hypothesis or primary outcome after seeing the data.

Statistical significance should no longer be used as a tool to screen what results are published and the evidence base would be in a better state if significance were given far less prominence [17]. The prominence given to statistical significance is ironic given the widespread misunderstandings of its true meaning [2, 12].

Limitations

We did not aim to develop a new statistical test for p-hacking or numerically compare the extent of p-hacking in: abstracts versus full-texts, or our sample of the published literature versus the sample with no p-hacking. Instead our aim was to present some simple plots that highlight the extent of the problem.

We included all confidence intervals, but we anticipate the biases would be even more striking if we were able to restrict the sample to the primary outcomes of interest [18]. This is because the statistical significance of the primary outcomes dictate the “success” or “failure” of the study, and less high-profile comparisons (e.g., between groups at baseline) are less likely to be p-hacked.

For peer review only

For peer review only - http://bmjopen.bmj.com/site/about/guidelines.xhtml

We have taken a broad look at the combined evidence and have not examined individual studies to show that they were biased, nor did we estimate the size of the bias. Instead, we aimed to show the extent of the bias across the literature using a simple graphical method. To our knowledge this is the first paper to examine the problem using confidence intervals and uses one of the largest sample sizes including results from both abstracts and full-texts. For us the graphs are a warning of the urgent need for action to improve research practices.

Footnotes

Funding statement: AGB was supported by the National Health and Medical Research Council grant number APP1117784.

Competing interests statement: None declared.

Data sharing statement: The dataset and code are freely available on *github* at https://github.com/jdwren/ASEC and https://github.com/agbarnett/intervals.

Contributors: AB and JW conceived the idea. JW wrote the programs to extract the data; AB wrote the analyses code. AB wrote the first draft of the paper with comments from JW. AB is responsible for the overall content as is the guarantor.

Figure legends

Figure 1: Empirical cumulative distributions for ratio confidence intervals from *Medline* abstracts and full papers. To be statistically significant, lower intervals need to be above 1 and upper intervals need to be under 1 (vertical lines). The x-axes are restricted to ratios between 0.25 and 4.

Figure 2: Empirical cumulative distributions for ratio confidence interval limits from a sample with no p-hacking or publication bias. "Negative" results were for associations with no known evidence of an association and "positive" studies were simulated using an increased risk. The x-axes are restricted to ratios between 0.25 and 4.

Figure 3: Empirical cumulative distributions for ratio confidence intervals from *Medline* abstracts in five year intervals. The x-axes are restricted to ratios between 0.25 and 4.

References

1. Gwendolyn B. Emerson, Winston J. Warme, Fredric M. Wolf, James D. Heckman, Richard A. Brand, and Seth S. Leopold. Testing for the presence of positive-outcome bias in peer review. *Archives of Internal Medicine*, 170(21), 2010.

For peer review only

For peer review only - http://bmjopen.bmj.com/site/about/guidelines.xhtml

2. S. Ziliak and D.N. McCloskey. *The Cult of Statistical Significance: How the Standard Error Costs Us Jobs, Justice, and Lives*. University of Michigan Press, 2008.

3. Uri Simonsohn, Leif D. Nelson, and Joseph P. Simmons. p-curve and effect size. *Perspectives on Psychological Science*, 9(6):666–681, 2014.

4. Megan L. Head, Luke Holman, Rob Lanfear, Andrew T. Kahn, and Michael D. Jennions. The extent and consequences of p-hacking in science. *PLOS Biology*, 13(3):1–15, 03 2015.

5. Andrew Gelman and Eric Loken. The garden of forking paths: Why multiple comparisons can be a problem, even when there is no "fishing expedition" or "p-hacking" and the research hypothesis was posited ahead of time. http://www.stat.columbia.edu/~gelman/research/unpublished/p_hacking.pdf 2013.

6. Aled M. Edwards, Ruth Isserlin, Gary D. Bader, Stephen V. Frye, Timothy M. Willson, and Frank H. Yu. Too many roads not taken. *Nature*, 470(7333):163–165, 2011.

7. David Chavalarias, Joshua David D. Wallach, Alvin Ho Ting H. Li, and John P. Ioannidis. Evolution of reporting P values in the biomedical literature, 1990–2015. *JAMA*, 315(11):1141–1148, 2016.

8. Constantin Georgescu and Jonathan D Wren. Algorithmic identification of discrepancies between published ratios and their reported confidence intervals and p-values. *Bioinformatics*, 34(10):1758–1766, 2017.

9. J. Ranstam. Why the p-value culture is bad and confidence intervals a better alternative. *Osteoarthritis and Cartilage*, 20(8):805–808, 2012.

10. Ronald L. Wasserstein and Nicole A. Lazar. The ASA's statement on p-values: Context, process, and purpose. *The American Statistician*, 70(2):129–133, 2016.

11. Dong Kyu Lee. Alternatives to p-value: confidence interval and effect size. *Korean Journal of Anesthesiology*, 69(6):555, 2016.

12. Steven Goodman. A dirty dozen: Twelve p-value misconceptions. *Seminars in Hematology*, 45(3):135–140, 2008.

13. Martijn J. Schuemie, Patrick B. Ryan, George Hripcsak, David Madigan, and Marc A. Suchard. Improving reproducibility by using high-throughput observational studies with empirical calibration. *Philosophical Transactions of the Royal Society A: Mathematical, Physical and Engineering Sciences*, 376(2128):20170356, 2018.

14. R Core Team. *R: A Language and Environment for Statistical Computing*. R Foundation for Statistical Computing, Vienna, Austria, 2018.

15. Fiona Fidler, Neil Thomason, Geoff Cumming, Sue Finch, and Joanna Leeman. Editors

For peer review only

For peer review only - http://bmjopen.bmj.com/site/about/guidelines.xhtml

can lead researchers to confidence intervals, but can't make them think. *Psychological Science*, 15(2):119–126, 2004.

16. Robert M. Kaplan and Veronica L. Irvin. Likelihood of null effects of large NHLBI clinical trials has increased over time. *PLOS ONE*, 10(8):e0132382, 2015.

17. Blakeley B. McShane and David Gal. Statistical significance and the dichotomization of evidence. *Journal of the American Statistical Association*, 112(519):885–895, 2017.

18. Uri Simonsohn. [41] falsely reassuring: Analyses of ALL p-values, http://datacolada.org/41 2015.

For peer review only

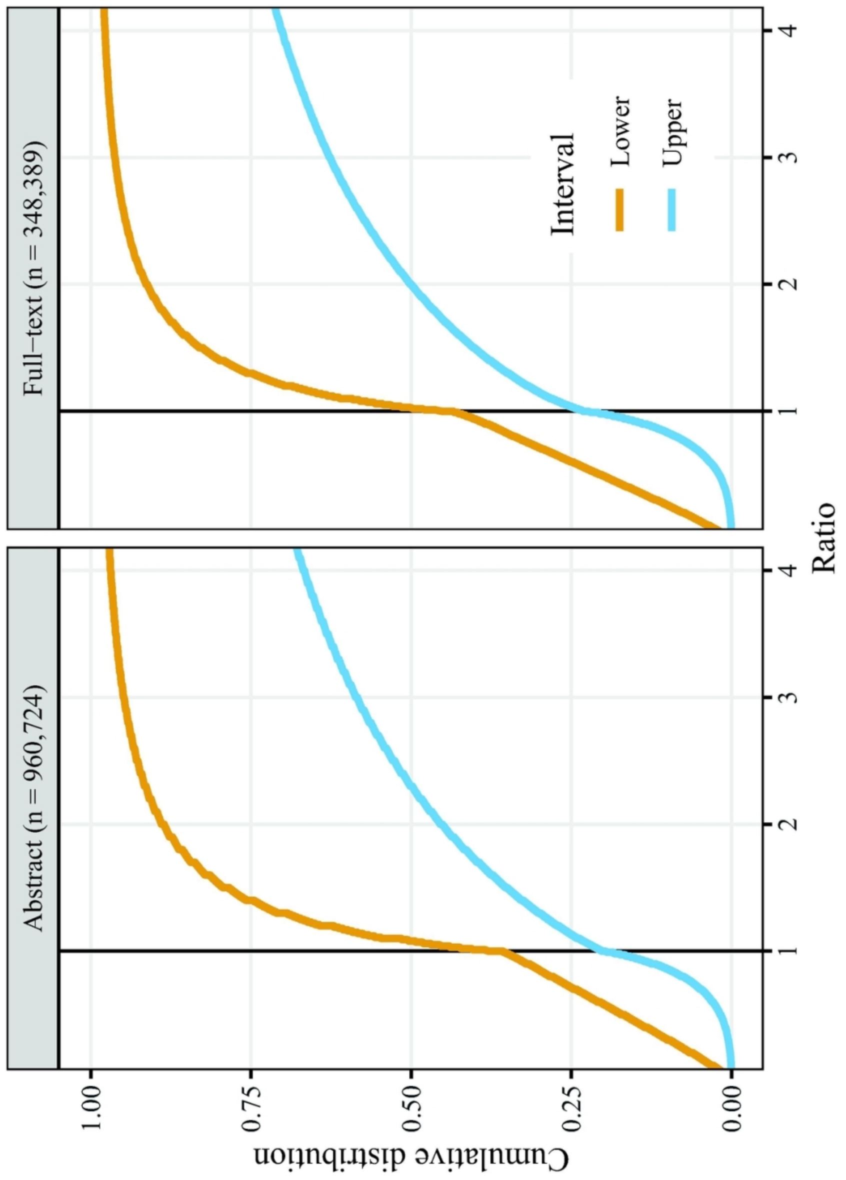

Empirical cumulative distributions for ratio confidence intervals from Medline abstracts and full papers. To be statistically significant, lower intervals need to be above 1 and upper intervals need to be under 1 (vertical lines). The x-axes are restricted to ratios between 0.25 and 4.

101x140mm (300 x 300 DPI)

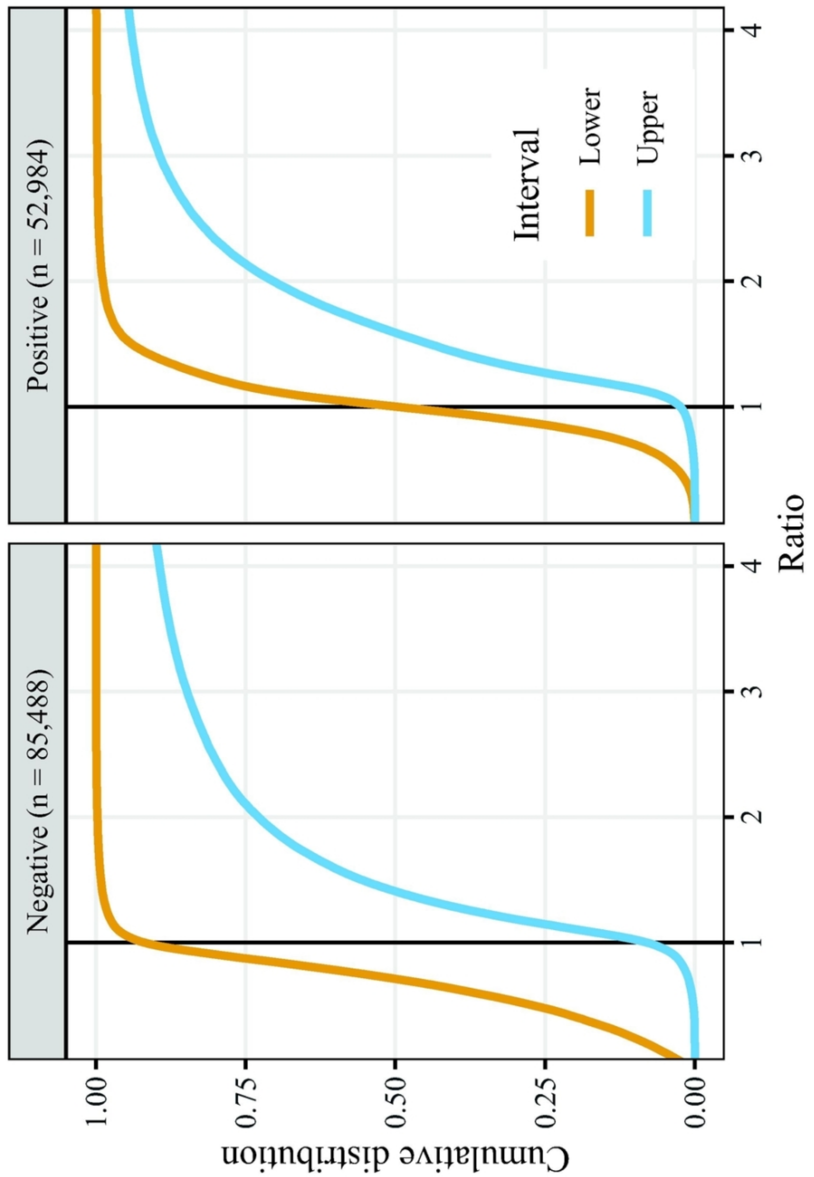

Empirical cumulative distributions for ratio confidence interval limits from a sample with no p-hacking or publication bias. “Negative” results were for associations with no known evidence of an association and “positive” studies were simulated using an increased risk. The x-axes are restricted to ratios between 0.25 and 4.

88x127mm (300 x 300 DPI)

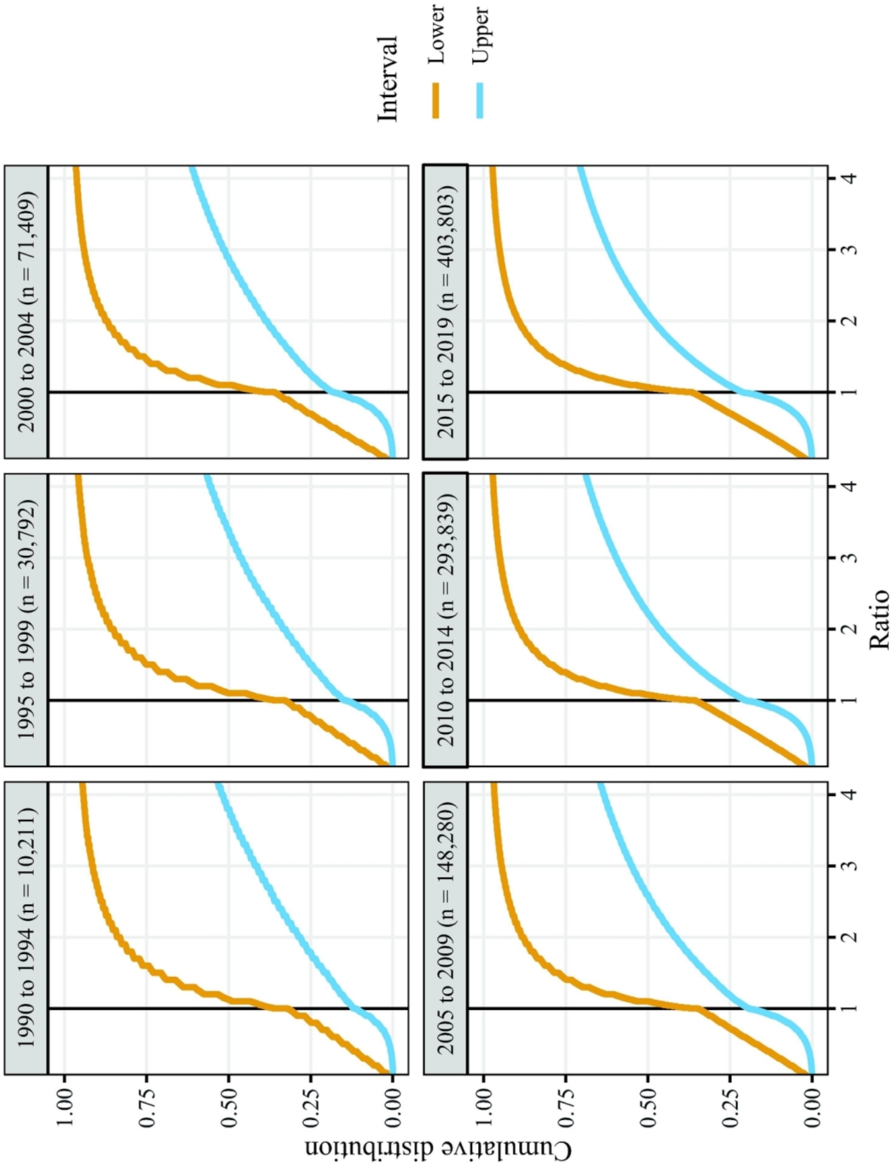

Empirical cumulative distributions for ratio confidence intervals from Medline abstracts in five year intervals. The x-axes are restricted to ratios between 0.25 and 4.

127x165mm (300 x 300 DPI)

Confidence intervals in health and medical journals show an implausible excess of statistically significant results

Adrian Barnett and Jonathan Wren

S1. Histograms

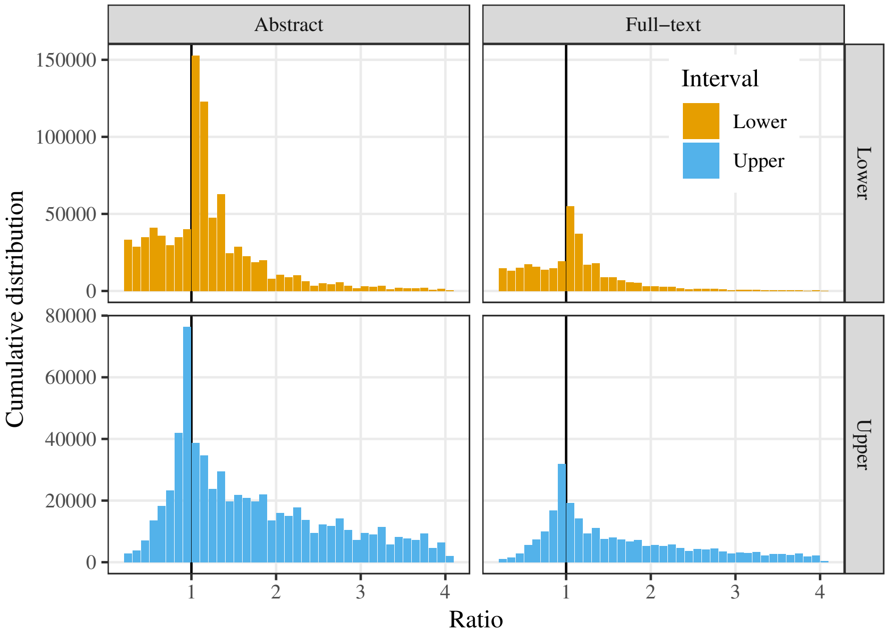

Figure S.1: Histograms for ratio confidence intervals from *Medline* abstracts and full-texts. The ratios are limited to 0.2 to 4.0.

The results in the figure above are an alternative version of the main results using a histogram rather than a cumulative density plot. We used binwidths of 0.1. The plot shows the dramatic rise in intervals just above 1 for the lower intervals, and rise in intervals below 1 for the upper intervals. The downside to using histograms is the requirement to select the binwidth and the plot's appearance does depend on this choice.

For peer review only - http://bmjopen.bmj.com/site/about/guidelines.xhtml

S2. Plots using a log-scale

In the results we focused on the key intervals close to 1 by restricting the intervals to between 0.25 and 4, which had the consequence of excluding 11% of lower intervals and 33% of upper intervals. The plot below uses a log-scale (base e) for the intervals which allows us to show ratios from 0.1 to 10. This plot excludes 4% of lower intervals and 15% of upper intervals.

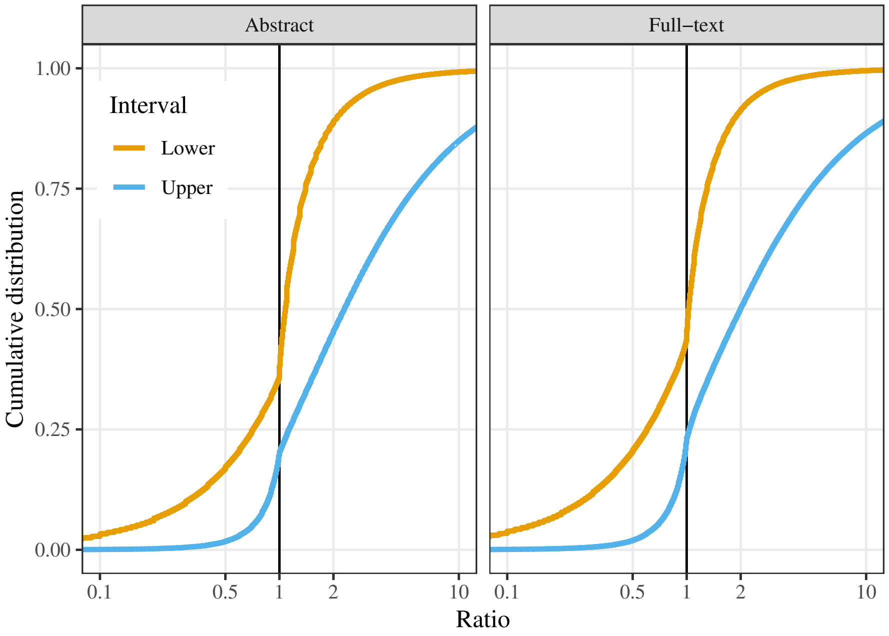

Figure S.2: Empirical cumulative distributions for ratio confidence intervals from *Medline* abstracts and full-texts. To be statistically significant, lower intervals need to be above 1 and upper intervals need to be under 1 (vertical lines). The x-axes are on a log-scale (base e).

The figure shows the same dramatic change close to 1 for the lower and upper intervals. One advantage of showing more of the data is that it confirms that the only discontinuity is at a ratio of 1.

For peer review only - http://bmjopen.bmj.com/site/about/guidelines.xhtml

S3. Intervals in a narrow range close to 1

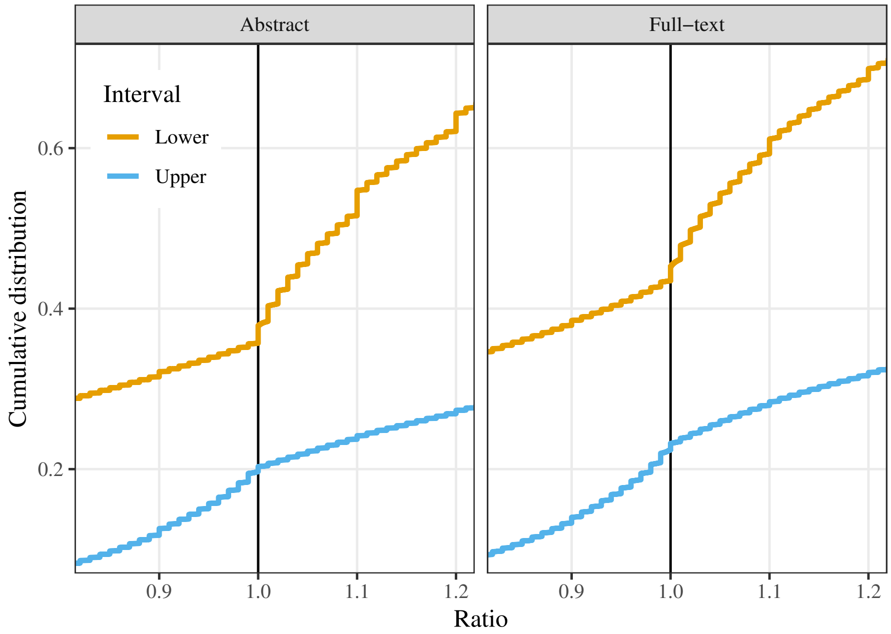

Figure S.3: Empirical cumulative distributions for ratio confidence intervals from *Medline* abstracts and full-texts. The ratios are limited to the narrow range of 0.83 to 1.2. The y-axes do not start at zero.

The plot above is an alternative look at the main results which examines those intervals close to 1. The x-axes are restricted to 0.83 to 1.2 and the y-axis are restricted to reduce white space. The plot includes 35% of all lower intervals and 20% of all upper intervals. The steps in the ratios are due to results being presented to 1 decimal place. The discontinuity in the cumulative numbers at 1 is still clearly visible and hence the changes shown in previous figures are not simply due to rounding.

For peer review only - http://bmjopen.bmj.com/site/about/guidelines.xhtml

S4. Full-text results over time

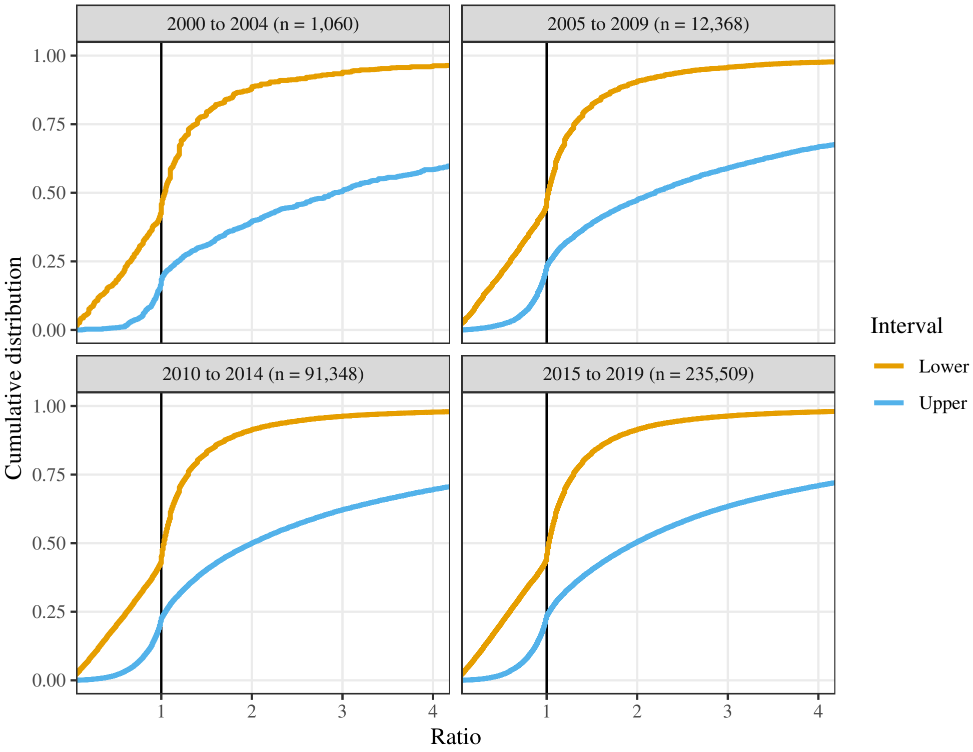

Figure S.4: Empirical cumulative distributions for ratio confidence intervals from full-texts in five year intervals. To be statistically significant, lower intervals need to be above 1 and upper intervals need to be under 1 (vertical lines).

The figure above is the complement to Figure 3 in the main paper, which examined the results over time from abstracts. This plot has a narrower time range because reasonable numbers of confidence intervals could only be extracted from the year 2000 onward. There was little change over time in the cumulative distributions, indicating little improvement in the bias for statistically significant results.

For peer review only - http://bmjopen.bmj.com/site/about/guidelines.xhtml

Reporting checklist for cross sectional study.

Based on the STROBE cross sectional guidelines.

Instructions to authors

Complete this checklist by entering the page numbers from your manuscript where readers will find each of the items listed below.

Your article may not currently address all the items on the checklist. Please modify your text to include the missing information. If you are certain that an item does not apply, please write "n/a" and provide a short explanation.

Upload your completed checklist as an extra file when you submit to a journal.

In your methods section, say that you used the STROBE cross sectional reporting guidelines, and cite them as:

von Elm E, Altman DG, Egger M, Pocock SJ, Gotzsche PC, Vandenbroucke JP. The Strengthening the Reporting of Observational Studies in Epidemiology (STROBE) Statement: guidelines for reporting observational studies.

		Reporting Item	Page Number
Title	#1a	Indicate the study's design with a commonly used term in the title or the abstract	1
Abstract	#1b	Provide in the abstract an informative and balanced summary of what was done and what was found	2
Background / rationale	#2	Explain the scientific background and rationale for the investigation being reported	2-3
Objectives	#3	State specific objectives, including any prespecified hypotheses	3
Study design	#4	Present key elements of study design early in the paper	3-4
Setting	#5	Describe the setting, locations, and relevant dates, including periods of recruitment, exposure, follow-up, and data collection	4
Eligibility criteria	#6a	Give the eligibility criteria, and the sources and methods of selection of participants.	4

For peer review only

	#7	Clearly define all outcomes, exposures, predictors, potential confounders, and effect modifiers. Give diagnostic criteria, if applicable	4-5
Data sources / measurement	#8	For each variable of interest give sources of data and details of methods of assessment (measurement). Describe comparability of assessment methods if there is more than one group. Give information separately for for exposed and unexposed groups if applicable.	4-5
Bias	#9	Describe any efforts to address potential sources of bias	n/a
Study size	#10	Explain how the study size was arrived at	See note 1
Quantitative variables	#11	Explain how quantitative variables were handled in the analyses. If applicable, describe which groupings were chosen, and why	4-5
Statistical methods	#12a	Describe all statistical methods, including those used to control for confounding	4-5
	#12b	Describe any methods used to examine subgroups and interactions	See note 2
	#12c	Explain how missing data were addressed	See note 3
	#12d	If applicable, describe analytical methods taking account of sampling strategy	See note 4
	#12e	Describe any sensitivity analyses	Supplementary file
Participants	#13a	Report numbers of individuals at each stage of study—eg numbers potentially eligible, examined for eligibility, confirmed eligible, included in the study, completing follow-up, and analysed. Give information separately for for exposed and unexposed groups if applicable.	5
	#13b	Give reasons for non-participation at each stage	See note 6
	#13c	Consider use of a flow diagram	See note 7
Descriptive data	#14a	Give characteristics of study participants (eg demographic, clinical, social) and information on exposures and potential confounders. Give information	See note 8

For peer review only

		separately for exposed and unexposed groups if applicable.	
	#14b	Indicate number of participants with missing data for each variable of interest	See note 9
Outcome data	#15	Report numbers of outcome events or summary measures. Give information separately for exposed and unexposed groups if applicable.	See note 10
Main results	#16a	Give unadjusted estimates and, if applicable, confounder-adjusted estimates and their precision (eg, 95% confidence interval). Make clear which confounders were adjusted for and why they were included	See note 11
	#16b	Report category boundaries when continuous variables were categorized	See note 12
	#16c	If relevant, consider translating estimates of relative risk into absolute risk for a meaningful time period	See note 13
Other analyses	#17	Report other analyses done—e.g., analyses of subgroups and interactions, and sensitivity analyses	See note 14
Key results	#18	Summarise key results with reference to study objectives	6
Limitations	#19	Discuss limitations of the study, taking into account sources of potential bias or imprecision. Discuss both direction and magnitude of any potential bias.	7
Interpretation	#20	Give a cautious overall interpretation considering objectives, limitations, multiplicity of analyses, results from similar studies, and other relevant evidence.	7
Generalisability	#21	Discuss the generalisability (external validity) of the study results	6
Funding	#22	Give the source of funding and the role of the funders for the present study and, if applicable, for the original study on which the present article is based	7

Author notes

1. n/a, used all available data from Medline

2. n/a, no subgroups or interactions

For peer review only

3. n/a, no missing data

4. n/a, was a census of all available data

5. n/a, no sensitivity analyses

6. n/a, no stages of participation

7. n/a, simple cross-sectional study with no stages

8. n/a, no real characteristics and there are there are no potential confounders or exposures

9. n/a, no missing data

10. n/a, no events

11. n/a, no statistical modelling

12. n/a, no boundaries used

13. n/a, no relative risk calculations

14. n/a, no additional analyses

The STROBE checklist is distributed under the terms of the Creative Commons Attribution License CC-BY. This checklist was completed on 28. February 2019 using https://www.goodreports.org/, a tool made by the EQUATOR Network in collaboration with Penelope.ai

For peer review only

BMJ Open

Confidence intervals in health and medical journals show an implausible excess of statistically significant results: an observational study

Journal:	*BMJ Open*
Manuscript ID	bmjopen-2019-032506.R1
Article Type:	Original research
Date Submitted by the Author:	28-Sep-2019
Complete List of Authors:	Barnett, Adrian; Queensland University of Technology, Institute of Health and Biomedical Innovation<br>Wren, Jonathan; Oklahoma Medical Research Foundation, Genes & Human Disease Research Program; University of Oklahoma Health Sciences Center, Department of Biochemistry and Molecular Biology
**Primary Subject Heading**:	Evidence based practice
Secondary Subject Heading:	Communication
Keywords:	p-values, statistical significance, confidence intervals, p-hacking, publication bias

SCHOLARONE™
Manuscripts

Title page

Title: Confidence intervals in health and medical journals show an implausible excess of statistically significant results: an observational study

Adrian G. Barnett [1]

Jonathan D. Wren [2,3]

[1] Institute of Health and Biomedical Innovation & School of Public Health and Social Work Queensland University of Technology, Australia

[2] Genes & Human Disease Research Program, Oklahoma Medical Research Foundation, Oklahoma City, OK, USA

[3] Department of Biochemistry and Molecular Biology, University of Oklahoma Health Sciences Center, Oklahoma City, OK, USA

Corresponding author's contact information:

Institute of Health and Biomedical Innovation & School of Public Health and Social Work

Queensland University of Technology

60 Musk Avenue

Kelvin Grove, Queensland, 4059

Australia

E-mail: a.barnett@qut.edu.au

Phone: ++61-7-3138 6010, Fax: ++61-7-3138 6030

Word count: 1760

For peer review only

For peer review only - http://bmjopen.bmj.com/site/about/guidelines.xhtml

Abstract

Objectives Previous research has shown clear biases in the distribution of published p-values, with an excess below the 0.05 threshold due to some combination of p-hacking and publication bias. We aimed to examine the bias for statistical significance using published confidence intervals.

Design Observational study.

Setting Papers published in *Medline* from 1976 to 2019.

Participants Over 968,000 confidence intervals extracted from abstracts and over 350,000 intervals extracted from the full-text.

Outcome measures Cumulative distributions of lower and upper confidence interval limits for ratio estimates.

Results We found an excess of statistically significant results with a glut of lower intervals just above 1 and upper intervals just below 1. These excesses have not improved in recent years. The excesses did not appear in a set of over 100,000 confidence intervals that were not subject to p-hacking or publication bias.

Conclusions The huge excesses of just statistically significant confidence intervals could not plausibly reflect unbiased hypothesis testing and reporting. Large improvements in research practice are needed to provide more results that better reflect the truth.

Keywords p-values, statistical significance, confidence intervals, p-hacking, publication bias

Strengths and limitations of this study:

- This is the first study to examine the bias towards statistical significance using confidence intervals instead of p-values.
- We used a very large sample of confidence intervals from both abstracts and full-texts.
- We used a simple graphical summary and did not develop a new test to detect p-hacking or examine differences in papers around the statistically significant threshold.
- We have taken a broad look at the combined evidence and have not examined individual studies to show that they were biased.

Introduction

Every health and medical researcher is aware of the importance of statistically significant

For peer review only

For peer review only - http://bmjopen.bmj.com/site/about/guidelines.xhtml

results, generally meaning a p-value less than 0.05. A study with statistically significant results is easier to publish in a journal [1] and is often perceived as having more validity and importance [2,3]. This can lead some researchers to strive for statistically significant results by re-analysing data purely to achieve a p-value under 0.05, known as "p-hacking" [4, 5]. This re-analyses can include changing the statistical assumptions, excluding data, or collecting additional data, but it does not include pre-specified secondary analyses. This striving for statistical significance is not always overt and can occur due to researchers making seemingly sensible scientific decisions [6]. It can also motivate researchers to carefully vet which hypotheses they pursue, selecting those they deem likely to yield significant results. For example, around 75% of scientific effort goes towards the 10% of genes already best characterized, suggesting widespread risk-aversion [7].

Journals are also somewhat responsible for the bias towards statistically significant results, partly because peer reviewers are more favourable to statistically significant results [1]. Journals depend on readership, and journal editors know that readers tend to be more interested in striking results (i.e., with a very low probability of an observation being the result of chance).

The combined "significance seeking" behaviour of researchers and journals has created a statistically implausible excess of published p-values just below the 0.05 threshold [1, 8–10]. This warped evidence undermines the purpose of evidence-based practice, as "negative" studies too often go unpublished and some published effect estimates are too large because p-hacking inflates effect sizes (by conditioning on statistically significant results). This biased evidence can harm the public's health when policy decisions are made using incomplete or overly-optimistic evidence.

Examining published p-values using the "p-curve" distribution can be used to indicate that p-hacking has occurred [3]. In this paper we show that the same excess occurs in confidence intervals, which are a recommended alternative to p-values for presenting results, and have been advocated as a way to avoid "bright-line" thinking at the 0.05 p-value threshold [3, 11–13]. We therefore take an alternative look at the already illustrated bias for statistical significance. However, we think this is worthwhile replication because this bias is a crucial problem for evidence-based medicine. Also using confidence intervals may lead to a wider understanding of the bias because researchers often misunderstand p-values [14]. We also show that the bias has not abated in recent years.

As a reminder, a p-value is the probability, under a specified model (usually null hypothesis

For peer review only

For peer review only - http://bmjopen.bmj.com/site/about/guidelines.xhtml

testing), of observing a test statistic as or more extreme than that observed in the data. A 95% confidence interval is a range that should contain the true value on 95% of occasions if the data generating process could be repeated many times. Most confidence intervals are given as 95% intervals, which correspond to a 0.05 p-value threshold.

Methods

We extracted ratio confidence intervals (e.g., odds, hazard and risk ratios) from the abstracts and full-texts of journals published between 1976 and 2019. The data were extracted using regular expressions, for full details see Georgescu and Wren [9]. The text-mining algorithm was designed to recognise the typical ways in which ratio estimates and confidence intervals are presented, and we selected ratios because they have a generally standard presentation meaning they can be extracted with a relatively high reliability. The results were independently validated using a separate text-mining algorithm developed by the first author (AGB). Random checks were made on approximately 5,000 abstracts and 500 full texts, as well as other checks on unusual results, e.g., extremely wide confidence intervals. The complete codes and data are available at https://github.com/jdwren/ASEC and https://github.com/agbarnett/intervals.

We excluded the 0.1% of lower intervals that were zero, assuming this was an error by the authors. We excluded the 0.7% of abstract intervals and 0.9% of full-text intervals where the point estimate was not within the confidence interval, as this meant either the interval or point estimate (or both) was incorrect.

We used a second set of published ratio estimates from health research based on thousands of analyses with no p-hacking or publication bias [15]. Using four large databases of insurance claims, the study examined all possible pairs of 17 treatments for depression using 22 outcomes. Risks were estimated using survival analysis, giving hazard ratios. Treatment–Outcome pairs were categorised as “negative” where there was no evidence in the literature of any association. “Positive” associations were created by simulating an increased risk with a hazard ratio of 1.5. Details are in the paper by Schuemie et al [15], the key point for our analysis is that we have a large sample of ratio confidence intervals from Treatment–Outcome pairs that are not subject to any “significance seeking” by researchers or journals.

Statistical methods

To summarise the intervals we plotted the cumulative empirical distribution of the lower and

For peer review only - http://bmjopen.bmj.com/site/about/guidelines.xhtml

upper confidence interval limits. We used the cumulative distribution because it useful for highlighting changes in the distribution, which is our key interest, and it does not require any user-defined tuning parameters. Histograms require the selection of a bin-width and density plots require user-defined smoothing parameters, which are subjective and will create different impressions depending on the choice made. We include histograms in the online supplement using a bin-width of 0.1, but our results focus on the cumulative distributions.

Our hypothesis was that there would be a large change in the intervals near 1, which is the commonly used null hypothesis of no difference on a ratio scale. A lower confidence interval limit for a ratio estimate that is greater than 1 would mean a statistically significant result, as would an upper interval limit below 1. To look for changes over time, we plotted separate cumulative densities in five year periods.

We used R version 3.6.0 for all analyses [16].

Patient and Public Involvement

No patients or members of the public were involved in this study.

Results

Our sample had over 968,000 intervals extracted from *Medline* abstracts from over 5,900 journals, and over 350,000 intervals extracted from the full-text from over 2,700 journals. For 11% of intervals the level of the confidence interval could not be determined, most often because it was not given. Where an interval could be determined, 99.7% were 95% confidence intervals, which corresponds to the commonly used p-value threshold of 0.05.

We found a clear and sudden change in the cumulative distribution of both lower and upper interval limits at the statistically significant threshold of 1 (Figure 1). There was a steep increase in the number of lower interval limits just above 1, meaning they were just above the threshold for statistical significance. Similarly there was a steep increase in the number of upper interval limits just below 1, and so just inside the statistically significant threshold. The jump at 1 appears slightly stronger (less smooth) in the abstract than the full-text and there are noticeably more lower intervals below 1 in the full-text, which suggests that the biases are stronger in the abstract than the full-text.

If there was no p-hacking and publication bias, the cumulative distributions would be smooth S-shapes with no jumps, and this is confirmed in Figure 2 using the data without these biases. The bias for statistically significant results in abstracts has persisted over time as shown by

For peer review only - http://bmjopen.bmj.com/site/about/guidelines.xhtml

the five-year plots in Figure 3. The plots also show the reduced use of rounded intervals over time, as the cumulative densities are smoother in later years.

The online supplement contain sensitivity analyses that: use wider and narrow ratio axes for the cumulative density plots; show histograms instead of cumulative densities; and show plots of the cumulative densities over time for the full-text. All the plots show the same striking changes in the number of intervals near 1.

Discussion

The strong bias for statistically significant results is clearly visible in the confidence intervals of a large sample of abstracts and full-texts from health and medical journals and has persisted for decades. This bias undermines the evidence base and impairs good decision making by giving a false picture of the effectiveness of treatments and procedures. Such biased evidence can harm patients and the public when it gets translated into policy and practice.

Confidence intervals have been promoted as a way of avoiding the simplistic bright-line thinking that often accompanies p-values. However, our results show that even though people are frequently reporting confidence intervals, many are still focusing on statistical significance. A push towards the greater use of confidence intervals in journals in place of p-values may therefore not solve the ongoing problem of bright-line thinking [17,18].

Large improvements in research practice are needed to improve the quality of evidence. Study results should be presented and published regardless of their statistical significance. This could be achieved by a wider uptake of study pre-registration and analysis plans [19], which reduces the potential for researchers to change their hypothesis or primary outcome after seeing the data.

Statistical significance should no longer be used as a tool to screen what results are published and the evidence base would be in a better state if significance were given far less prominence [20]. The prominence given to statistical significance is ironic given the widespread misunderstandings of its true meaning [2, 14].

Limitations

We did not aim to develop a new statistical test for p-hacking or numerically compare the extent of p-hacking in: abstracts versus full-texts, or our sample of the published literature versus the sample with no p-hacking. Instead our aim was to present some simple plots that

For peer review only

highlight the extent of the problem.

We included all confidence intervals, but it is possible the biases would be even more striking if we were able to restrict the sample to the primary outcomes of interest [21]. This is because the statistical significance of the primary outcomes dictate the "success" or "failure" of the study, and less high-profile comparisons (e.g., between groups at baseline) are less likely to be p-hacked.

We have taken a broad look at the combined evidence and have not examined individual studies to show that they were biased, nor did we estimate the size of the bias. Instead, we aimed to show the extent of the bias across the literature using a simple graphical method. To our knowledge this is the first paper to examine the problem using confidence intervals and uses one of the largest sample sizes including results from both abstracts and full-texts. For us the graphs are a warning of the urgent need for action to improve research practices.

Footnotes

Funding statement: AGB was supported by the National Health and Medical Research Council grant number APP1117784.

Competing interests statement: None declared.

Data sharing statement: The dataset and code are freely available on *github* at https://github.com/jdwren/ASEC and https://github.com/agbarnett/intervals.

Contributors: AB and JW conceived the idea. JW wrote the programs to extract the data; AB wrote the analyses code. AB wrote the first draft of the paper with comments from JW. AB is responsible for the overall content as is the guarantor.

Figure legends

Figure 1: Empirical cumulative distributions for ratio confidence intervals from *Medline* abstracts and full papers. To be statistically significant, lower intervals need to be above 1 and upper intervals need to be under 1 (vertical lines). The x-axes are restricted to ratios between 0.25 and 4.

Figure 2: Empirical cumulative distributions for ratio confidence interval limits from a sample with no p-hacking or publication bias. "Negative" results were for associations with no known evidence of an association and "positive" studies were simulated using an increased risk. The x-axes are restricted to ratios between 0.25 and 4.

Figure 3: Empirical cumulative distributions for ratio confidence intervals from *Medline*

For peer review only - http://bmjopen.bmj.com/site/about/guidelines.xhtml

abstracts in five year intervals. The x-axes are restricted to ratios between 0.25 and 4.

References

1. Gwendolyn B. Emerson, Winston J. Warme, Fredric M. Wolf, James D. Heckman, Richard A. Brand, and Seth S. Leopold. Testing for the presence of positive-outcome bias in peer review. *Archives of Internal Medicine*, 170(21), 2010.
2. S. Ziliak and D.N. McCloskey. *Chapter 14: Medicine Seeks a Magic Pill*. In *The Cult of Statistical Significance: How the Standard Error Costs Us Jobs, Justice, and Lives*. University of Michigan Press, 2008.
3. Robert J. Calin-Jageman & Geoff Cumming (2019) The New Statistics for Better Science: Ask How Much, How Uncertain, and What Else Is Known, *The American Statistician*, 73:sup1, 271–280, DOI: 10.1080/00031305.2018.1518266
4. Uri Simonsohn, Leif D. Nelson, and Joseph P. Simmons. p-curve and effect size. *Perspectives on Psychological Science*, 9(6):666–681, 2014.
5. Megan L. Head, Luke Holman, Rob Lanfear, Andrew T. Kahn, and Michael D. Jennions. The extent and consequences of p-hacking in science. *PLOS Biology*, 13(3):1–15, 03 2015.
6. Andrew Gelman and Eric Loken. The garden of forking paths: Why multiple comparisons can be a problem, even when there is no "fishing expedition" or "p-hacking" and the research hypothesis was posited ahead of time. http://www.stat.columbia.edu/~gelman/research/unpublished/p_hacking.pdf 2013.
7. Aled M. Edwards, Ruth Isserlin, Gary D. Bader, Stephen V. Frye, Timothy M. Willson, and Frank H. Yu. Too many roads not taken. *Nature*, 470(7333):163–165, 2011.
8. David Chavalarias, Joshua David D. Wallach, Alvin Ho Ting H. Li, and John P. Ioannidis. Evolution of reporting P values in the biomedical literature, 1990–2015. *JAMA*, 315(11):1141–1148, 2016.
9. Constantin Georgescu and Jonathan D Wren. Algorithmic identification of discrepancies between published ratios and their reported confidence intervals and p-values. *Bioinformatics*, 34(10):1758–1766, 2017.
10. Edwin Kagereki, Joseph Gakonyo & Hazel Simila. Significance bias: an empirical evaluation of the oral health literature. *BMC Oral Health*, 16: 53, 2016.
11. J. Ranstam. Why the p-value culture is bad and confidence intervals a better alternative. *Osteoarthritis and Cartilage*, 20(8):805–808, 2012.
12. Ronald L. Wasserstein and Nicole A. Lazar. The ASA's statement on p-values: Context,

For peer review only - http://bmjopen.bmj.com/site/about/guidelines.xhtml

process, and purpose. *The American Statistician*, 70(2):129–133, 2016.

13. Dong Kyu Lee. Alternatives to p-value: confidence interval and effect size. *Korean Journal of Anesthesiology*, 69(6):555, 2016.

14. Steven Goodman. A dirty dozen: Twelve p-value misconceptions. *Seminars in Hematology*, 45(3):135–140, 2008.

15. Martijn J. Schuemie, Patrick B. Ryan, George Hripcsak, David Madigan, and Marc A. Suchard. Improving reproducibility by using high-throughput observational studies with empirical calibration. *Philosophical Transactions of the Royal Society A: Mathematical, Physical and Engineering Sciences*, 376(2128):20170356, 2018.

16. R Core Team. *R: A Language and Environment for Statistical Computing*. R Foundation for Statistical Computing, Vienna, Austria, 2018.

17. Fiona Fidler, Neil Thomason, Geoff Cumming, Sue Finch, and Joanna Leeman. Editors can lead researchers to confidence intervals, but can't make them think. *Psychological Science*, 15(2):119–126, 2004.

18. Steven N. Goodman. Why is Getting Rid of P-Values So Hard? Musings on Science and Statistics, *The American Statistician*, 73:sup1, 26-30, 2019. DOI: 10.1080/00031305.2018.1558111

19. Robert M. Kaplan and Veronica L. Irvin. Likelihood of null effects of large NHLBI clinical trials has increased over time. *PLOS ONE*, 10(8):e0132382, 2015.

20. Blakeley B. McShane and David Gal. Statistical significance and the dichotomization of evidence. *Journal of the American Statistical Association*, 112(519):885–895, 2017.

21. Uri Simonsohn. [41] falsely reassuring: Analyses of ALL p-values, http://datacolada.org/41 2015.

For peer review only

For peer review only - http://bmjopen.bmj.com/site/about/guidelines.xhtml

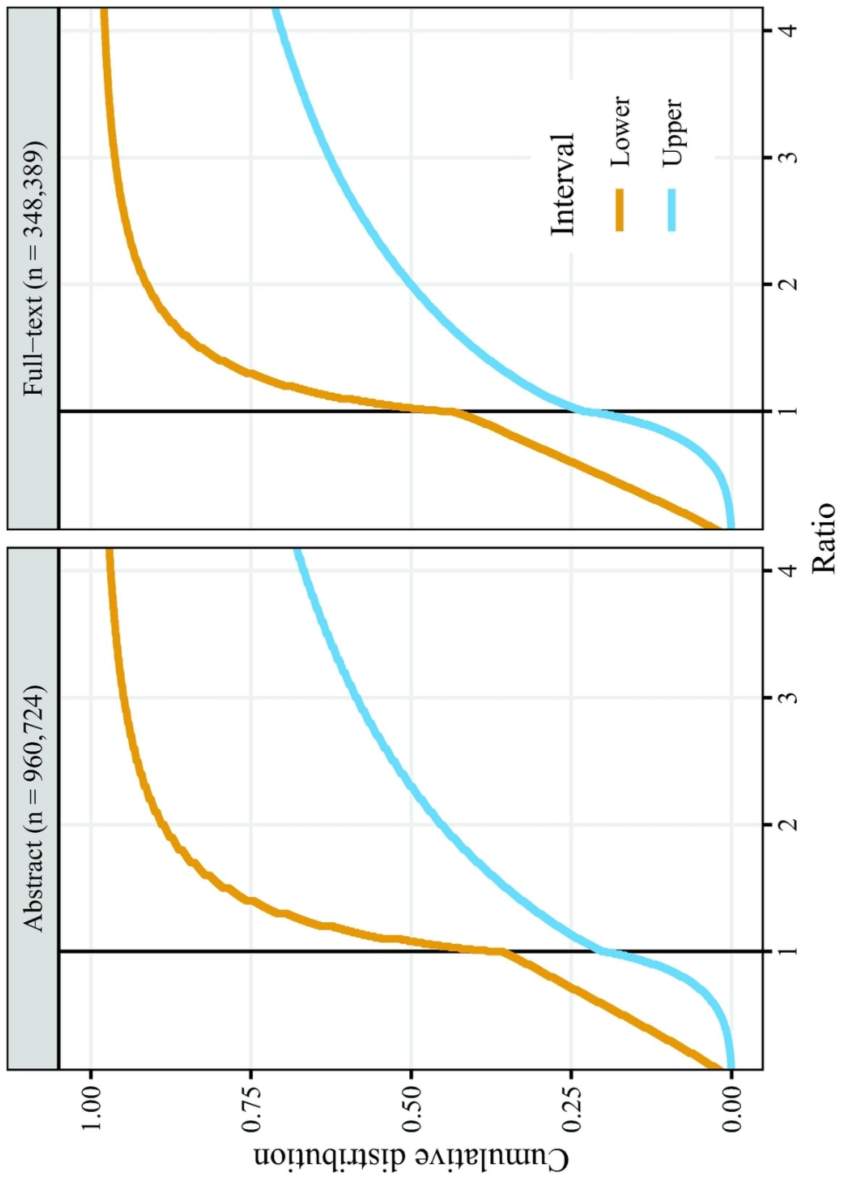

Empirical cumulative distributions for ratio confidence intervals from Medline abstracts and full papers. To be statistically significant, lower intervals need to be above 1 and upper intervals need to be under 1 (vertical lines). The x-axes are restricted to ratios between 0.25 and 4.

101x140mm (300 x 300 DPI)

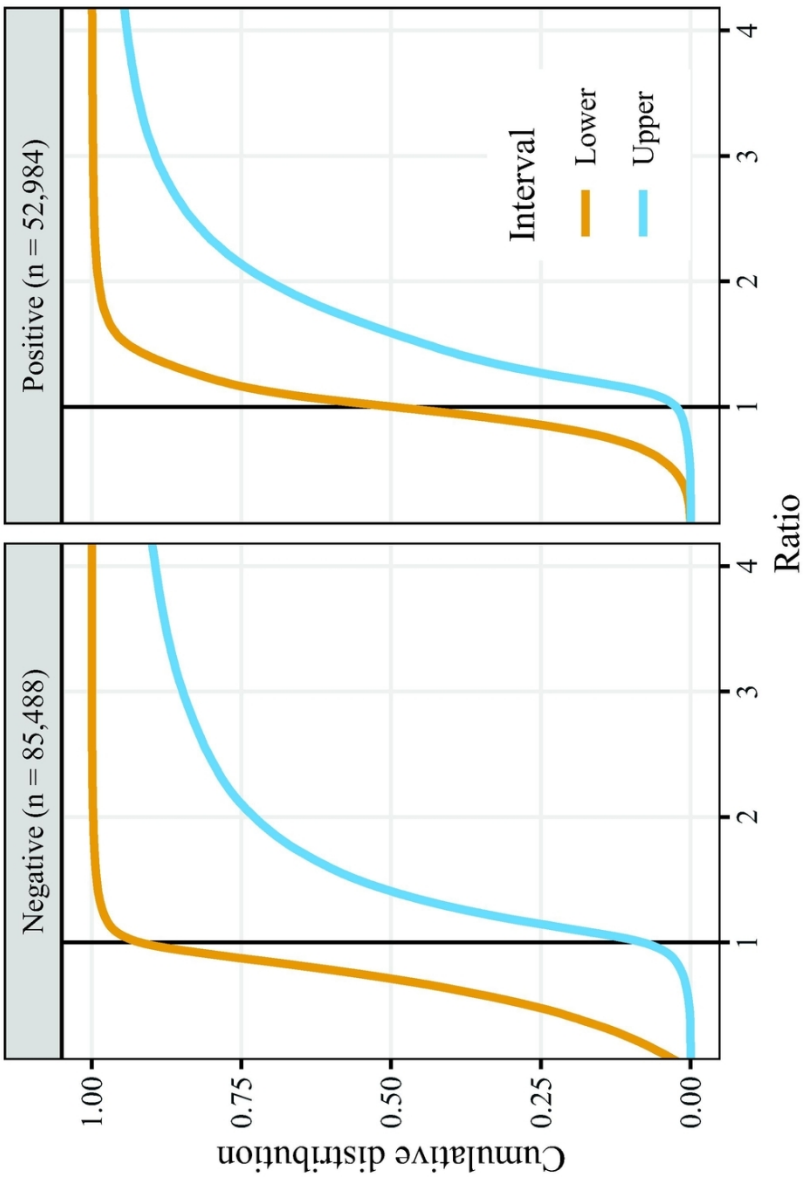

Empirical cumulative distributions for ratio confidence interval limits from a sample with no p-hacking or publication bias. “Negative” results were for associations with no known evidence of an association and “positive” studies were simulated using an increased risk. The x-axes are restricted to ratios between 0.25 and 4.

88x127mm (300 x 300 DPI)

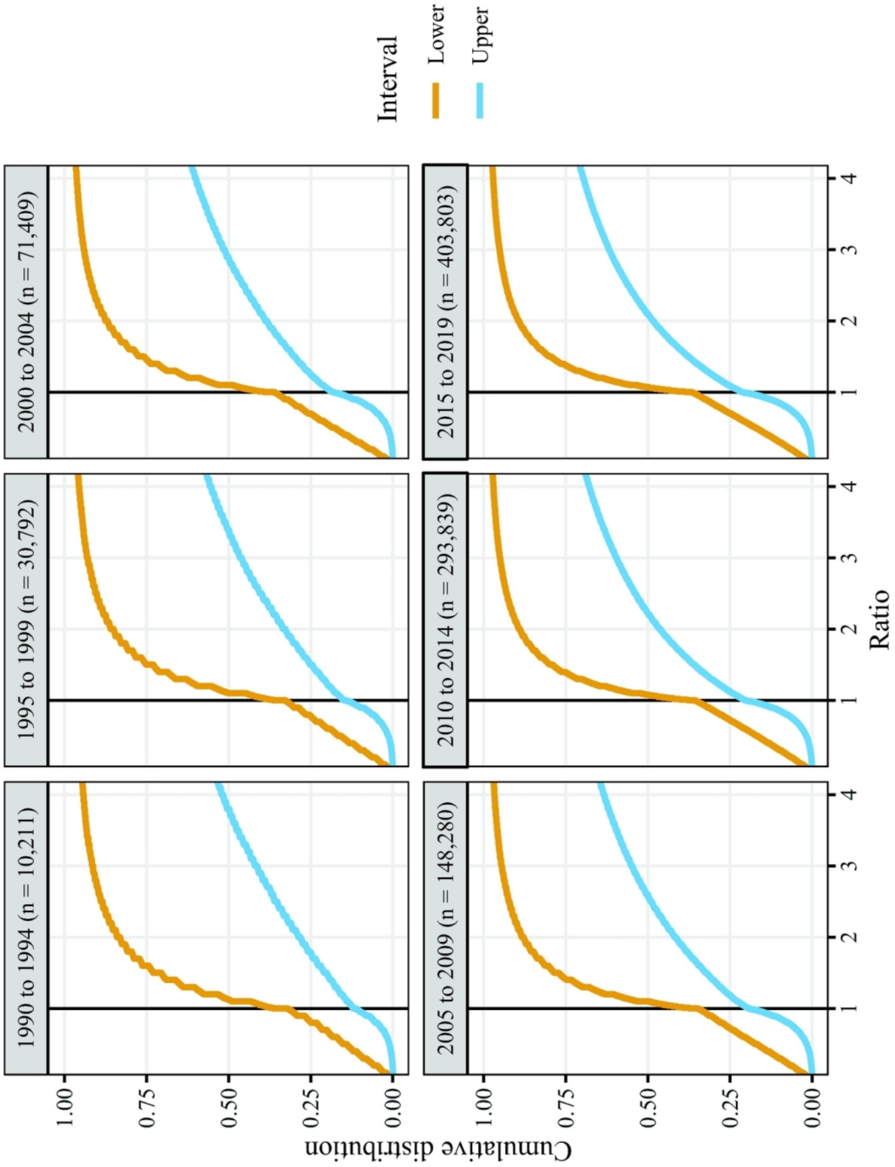

Empirical cumulative distributions for ratio confidence intervals from Medline abstracts in five year intervals. The x-axes are restricted to ratios between 0.25 and 4.

127x165mm (300 x 300 DPI)

Confidence intervals in health and medical journals show an implausible excess of statistically significant results

Adrian Barnett and Jonathan Wren

S1. Histograms

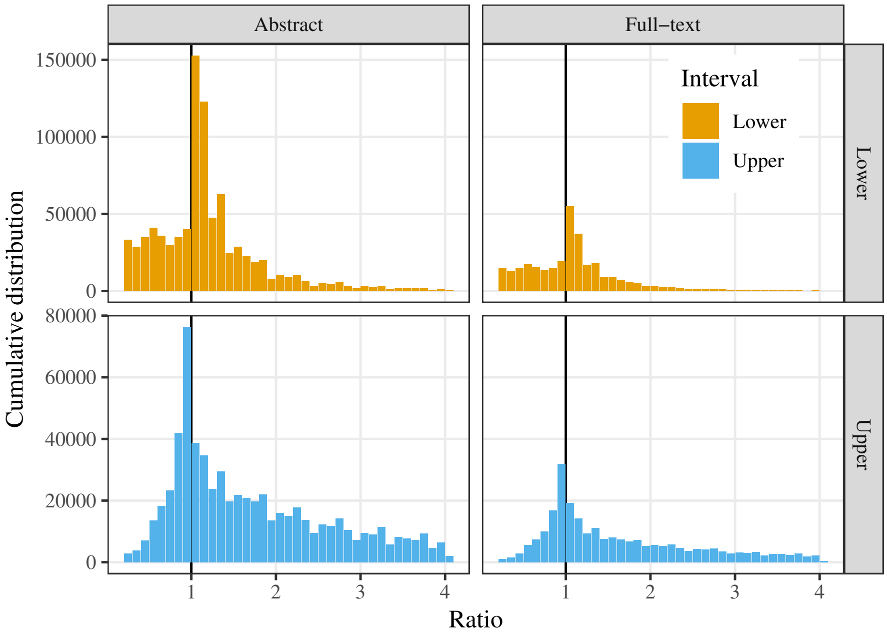

Figure S.1: Histograms for ratio confidence intervals from *Medline* abstracts and full-texts. The ratios are limited to 0.2 to 4.0.

The results in the figure above are an alternative version of the main results using a histogram rather than a cumulative density plot. We used binwidths of 0.1. The plot shows the dramatic rise in intervals just above 1 for the lower intervals, and rise in intervals below 1 for the upper intervals. The downside to using histograms is the requirement to select the binwidth and the plot's appearance does depend on this choice.

For peer review only - http://bmjopen.bmj.com/site/about/guidelines.xhtml

S2. Plots using a log-scale

In the results we focused on the key intervals close to 1 by restricting the intervals to between 0.25 and 4, which had the consequence of excluding 11% of lower intervals and 33% of upper intervals. The plot below uses a log-scale (base e) for the intervals which allows us to show ratios from 0.1 to 10. This plot excludes 4% of lower intervals and 15% of upper intervals.

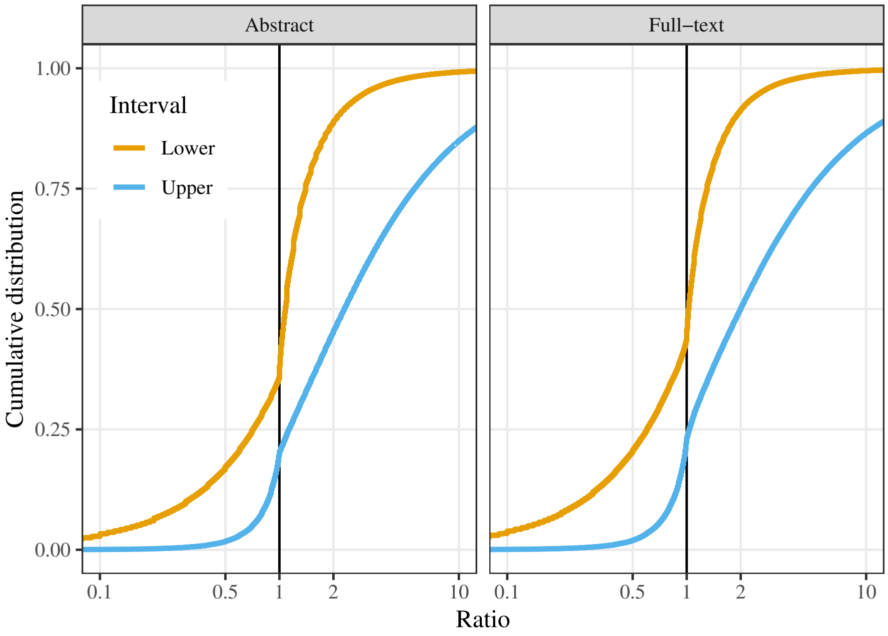

Figure S.2: Empirical cumulative distributions for ratio confidence intervals from *Medline* abstracts and full-texts. To be statistically significant, lower intervals need to be above 1 and upper intervals need to be under 1 (vertical lines). The x-axes are on a log-scale (base e).

The figure shows the same dramatic change close to 1 for the lower and upper intervals. One advantage of showing more of the data is that it confirms that the only discontinuity is at a ratio of 1.

For peer review only - http://bmjopen.bmj.com/site/about/guidelines.xhtml

S3. Intervals in a narrow range close to 1

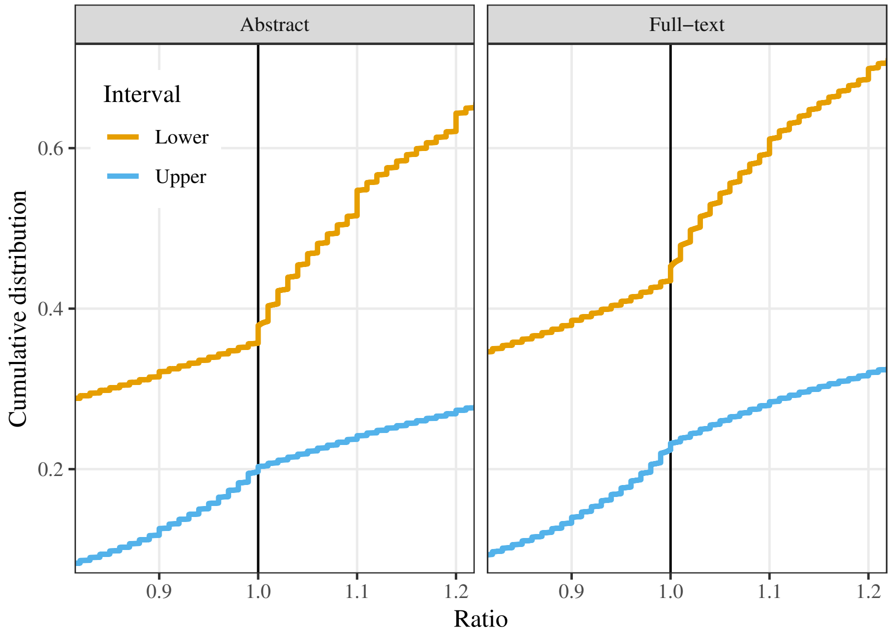

Figure S.3: Empirical cumulative distributions for ratio confidence intervals from *Medline* abstracts and full-texts. The ratios are limited to the narrow range of 0.83 to 1.2. The y-axes do not start at zero.

The plot above is an alternative look at the main results which examines those intervals close to 1. The x-axes are restricted to 0.83 to 1.2 and the y-axis are restricted to reduce white space. The plot includes 35% of all lower intervals and 20% of all upper intervals. The steps in the ratios are due to results being presented to 1 decimal place. The discontinuity in the cumulative numbers at 1 is still clearly visible and hence the changes shown in previous figures are not simply due to rounding.

For peer review only - http://bmjopen.bmj.com/site/about/guidelines.xhtml

S4. Full-text results over time

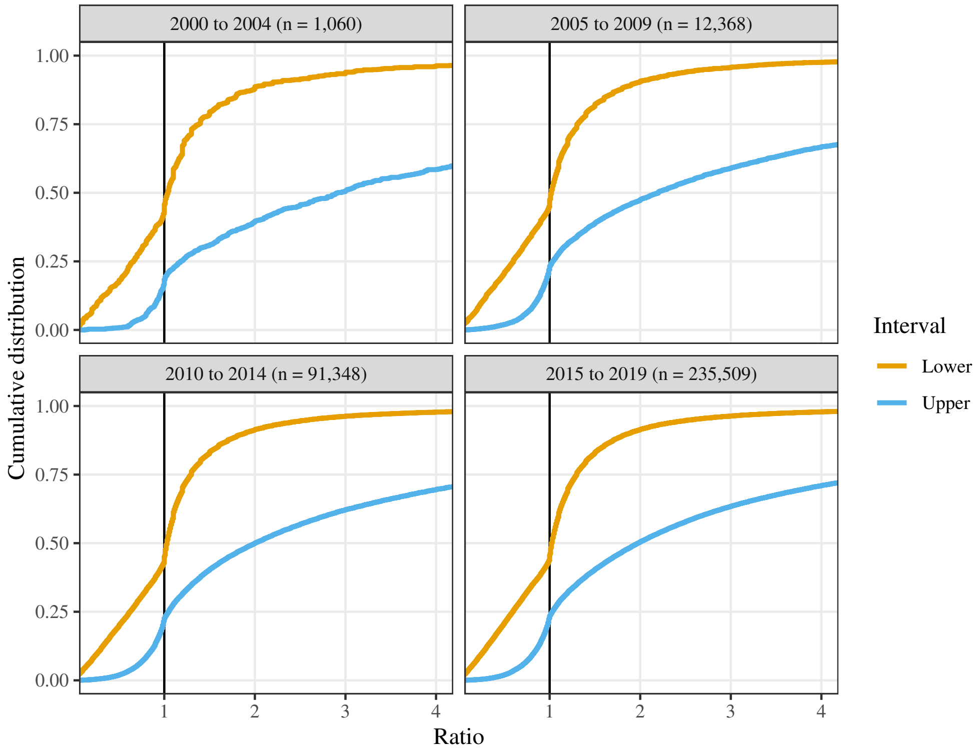

Figure S.4: Empirical cumulative distributions for ratio confidence intervals from full-texts in five year intervals. To be statistically significant, lower intervals need to be above 1 and upper intervals need to be under 1 (vertical lines).

The figure above is the complement to Figure 3 in the main paper, which examined the results over time from abstracts. This plot has a narrower time range because reasonable numbers of confidence intervals could only be extracted from the year 2000 onward. There was little change over time in the cumulative distributions, indicating little improvement in the bias for statistically significant results.

For peer review only - http://bmjopen.bmj.com/site/about/guidelines.xhtml

Reporting checklist for cross sectional study.

Based on the STROBE cross sectional guidelines.

Instructions to authors

Complete this checklist by entering the page numbers from your manuscript where readers will find each of the items listed below.

Your article may not currently address all the items on the checklist. Please modify your text to include the missing information. If you are certain that an item does not apply, please write "n/a" and provide a short explanation.

Upload your completed checklist as an extra file when you submit to a journal.

In your methods section, say that you used the STROBE cross sectional reporting guidelines, and cite them as:

von Elm E, Altman DG, Egger M, Pocock SJ, Gotzsche PC, Vandenbroucke JP. The Strengthening the Reporting of Observational Studies in Epidemiology (STROBE) Statement: guidelines for reporting observational studies.

		Reporting Item	Page Number
Title	#1a	Indicate the study's design with a commonly used term in the title or the abstract	1
Abstract	#1b	Provide in the abstract an informative and balanced summary of what was done and what was found	2
Background / rationale	#2	Explain the scientific background and rationale for the investigation being reported	2-3
Objectives	#3	State specific objectives, including any prespecified hypotheses	3
Study design	#4	Present key elements of study design early in the paper	3-4
Setting	#5	Describe the setting, locations, and relevant dates, including periods of recruitment, exposure, follow-up, and data collection	4
Eligibility criteria	#6a	Give the eligibility criteria, and the sources and methods of selection of participants.	4

For peer review only

	#7	Clearly define all outcomes, exposures, predictors, potential confounders, and effect modifiers. Give diagnostic criteria, if applicable	4-5
Data sources / measurement	#8	For each variable of interest give sources of data and details of methods of assessment (measurement). Describe comparability of assessment methods if there is more than one group. Give information separately for for exposed and unexposed groups if applicable.	4-5
Bias	#9	Describe any efforts to address potential sources of bias	n/a
Study size	#10	Explain how the study size was arrived at	See note 1
Quantitative variables	#11	Explain how quantitative variables were handled in the analyses. If applicable, describe which groupings were chosen, and why	4-5
Statistical methods	#12a	Describe all statistical methods, including those used to control for confounding	4-5
	#12b	Describe any methods used to examine subgroups and interactions	See note 2
	#12c	Explain how missing data were addressed	See note 3
	#12d	If applicable, describe analytical methods taking account of sampling strategy	See note 4
	#12e	Describe any sensitivity analyses	Supplementary file
Participants	#13a	Report numbers of individuals at each stage of study—eg numbers potentially eligible, examined for eligibility, confirmed eligible, included in the study, completing follow-up, and analysed. Give information separately for for exposed and unexposed groups if applicable.	5
	#13b	Give reasons for non-participation at each stage	See note 6
	#13c	Consider use of a flow diagram	See note 7
Descriptive data	#14a	Give characteristics of study participants (eg demographic, clinical, social) and information on exposures and potential confounders. Give information	See note 8

For peer review only

		separately for exposed and unexposed groups if applicable.	
	#14b	Indicate number of participants with missing data for each variable of interest	See note 9
Outcome data	#15	Report numbers of outcome events or summary measures. Give information separately for exposed and unexposed groups if applicable.	See note 10
Main results	#16a	Give unadjusted estimates and, if applicable, confounder-adjusted estimates and their precision (eg, 95% confidence interval). Make clear which confounders were adjusted for and why they were included	See note 11
	#16b	Report category boundaries when continuous variables were categorized	See note 12
	#16c	If relevant, consider translating estimates of relative risk into absolute risk for a meaningful time period	See note 13
Other analyses	#17	Report other analyses done—e.g., analyses of subgroups and interactions, and sensitivity analyses	See note 14
Key results	#18	Summarise key results with reference to study objectives	6
Limitations	#19	Discuss limitations of the study, taking into account sources of potential bias or imprecision. Discuss both direction and magnitude of any potential bias.	7
Interpretation	#20	Give a cautious overall interpretation considering objectives, limitations, multiplicity of analyses, results from similar studies, and other relevant evidence.	7
Generalisability	#21	Discuss the generalisability (external validity) of the study results	6
Funding	#22	Give the source of funding and the role of the funders for the present study and, if applicable, for the original study on which the present article is based	7

Author notes

1. n/a, used all available data from Medline

2. n/a, no subgroups or interactions

For peer review only

3. n/a, no missing data

4. n/a, was a census of all available data

5. n/a, no sensitivity analyses

6. n/a, no stages of participation

7. n/a, simple cross-sectional study with no stages

8. n/a, no real characteristics and there are there are no potential confounders or exposures

9. n/a, no missing data

10. n/a, no events

11. n/a, no statistical modelling

12. n/a, no boundaries used

13. n/a, no relative risk calculations

14. n/a, no additional analyses

The STROBE checklist is distributed under the terms of the Creative Commons Attribution License CC-BY. This checklist was completed on 28. February 2019 using https://www.goodreports.org/, a tool made by the EQUATOR Network in collaboration with Penelope.ai

For peer review only

BMJ Open

An examination of confidence intervals in health and medical journals from 1976 to 2019: an observational study

Journal:	*BMJ Open*
Manuscript ID	bmjopen-2019-032506.R2
Article Type:	Original research
Date Submitted by the Author:	04-Nov-2019
Complete List of Authors:	Barnett, Adrian; Queensland University of Technology, Institute of Health and Biomedical Innovation<br>Wren, Jonathan; Oklahoma Medical Research Foundation, Genes & Human Disease Research Program; University of Oklahoma Health Sciences Center, Department of Biochemistry and Molecular Biology
**Primary Subject Heading**:	Evidence based practice
Secondary Subject Heading:	Communication
Keywords:	p-values, statistical significance, confidence intervals, p-hacking, publication bias

SCHOLARONE™
Manuscripts

Title page

Title: An examination of confidence intervals in health and medical journals from 1976 to 2019: an observational study

Adrian G. Barnett [1]

Jonathan D. Wren [2,3]

[1] Institute of Health and Biomedical Innovation & School of Public Health and Social Work Queensland University of Technology, Australia

[2] Arthritis and Clinical Immunology Research Program, Division of Genomics and Data Sciences, Oklahoma Medical Research Foundation, Oklahoma City, OK, USA

[3] Department of Biochemistry and Molecular Biology, University of Oklahoma Health Sciences Center, Oklahoma City, OK, USA

Corresponding author's contact information:

Institute of Health and Biomedical Innovation & School of Public Health and Social Work

Queensland University of Technology

60 Musk Avenue

Kelvin Grove, Queensland, 4059

Australia

E-mail: a.barnett@qut.edu.au

Phone: ++61-7-3138 6010, Fax: ++61-7-3138 6030

Word count: 1661

For peer review only

Abstract

Objectives Previous research has shown clear biases in the distribution of published p-values, with an excess below the 0.05 threshold due to a combination of p-hacking and publication bias. We aimed to examine the bias for statistical significance using published confidence intervals.

Design Observational study.

Setting Papers published in *Medline* since 1976.

Participants Over 968,000 confidence intervals extracted from abstracts and over 350,000 intervals extracted from the full-text.

Outcome measures Cumulative distributions of lower and upper confidence interval limits for ratio estimates.

Results We found an excess of statistically significant results with a glut of lower intervals just above 1 and upper intervals just below 1. These excesses have not improved in recent years. The excesses did not appear in a set of over 100,000 confidence intervals that were not subject to p-hacking or publication bias.

Conclusions The huge excesses of published confidence intervals that are just below the statistically significant threshold are not statistically plausible. Large improvements in research practice are needed to provide more results that better reflect the truth.

Keywords p-values, statistical significance, confidence intervals, p-hacking, publication bias

Strengths and limitations of this study:

- This is the first study to examine the bias towards statistical significance using confidence intervals instead of p-values.
- We used a very large sample of confidence intervals from both abstracts and full-texts.
- We used a simple graphical summary and did not develop a new test to detect p-hacking or examine differences in papers around the statistically significant threshold.
- We have taken a broad look at the combined evidence and have not examined individual studies to show that they were biased.

Introduction

Every health and medical researcher is aware of the importance of statistically significant results, generally meaning a p-value less than 0.05. A study with statistically significant

For peer review only

For peer review only - http://bmjopen.bmj.com/site/about/guidelines.xhtml

results is easier to publish in a journal [1] and statistical significance can be mistaken for study validity and importance [2]. This can lead some researchers to strive for statistically significant results by re-analysing data to get a p-value under 0.05, known as “p-hacking” [3, 4]. This striving for statistical significance is not always overt and can occur due to researchers making seemingly sensible scientific decisions [5]. It can also motivate researchers to carefully vet which hypotheses they pursue, selecting those they deem likely to yield significant results. For example, around 75% of scientific effort goes towards the 10% of genes already best characterized, suggesting widespread risk-aversion [6].

Journals are also somewhat responsible for the bias towards statistically significant results. Journals depend on readership, and journal editors know that readers tend to be more interested in striking results.

The combined “significance seeking” behaviour of researchers and journals has created a statistically implausible excess of published p-values just below the 0.05 threshold [1, 7, 8]. This warped evidence undermines the purpose of evidence-based practice, as “negative” studies too often go unpublished and some published effect estimates are too large because p-hacking inflates effect sizes (by conditioning on statistically significant results). This biased evidence can harm the public’s health when policy decisions are made using incomplete or overly-optimistic evidence.

Examining published p-values using the “p-curve” distribution can be used to indicate that p-hacking has occurred [3]. In this paper we show that the same excess occurs in confidence intervals, which are a recommended alternative to p-values for presenting results, and have been advocated as a way to avoid “bright-line” thinking at the 0.05 p-value threshold [9, 10, 11]. We therefore take an alternative look at the already illustrated bias for statistical significance. However, we think this is worthwhile replication because this bias is a crucial problem for evidence-based medicine. Also using confidence intervals may lead to a wider understanding of the bias because researchers often misunderstand p-values [12]. We also show that the bias has not abated in recent years.

As a reminder, a p-value is the probability, under a specified model, of observing a test statistic as or more extreme than that observed in the data. A 95% confidence interval is a range that should contain the true value on 95% of occasions if the data generating process could be repeated many times. Most confidence intervals are given as 95% intervals, which correspond to a 0.05 p-value threshold.

For peer review only

For peer review only - http://bmjopen.bmj.com/site/about/guidelines.xhtml

Methods

We extracted ratio confidence intervals from the abstracts and full-texts of journals using regular expressions, for full details see Georgescu and Wren [8]. The text-mining algorithm was designed to recognise the typical ways in which ratio estimates (e.g., odds, hazard and risk ratios) and confidence intervals are presented. The results were independently validated using a separate text-mining algorithm developed by the first author (AGB). Random checks were made on approximately 5,000 abstracts and 500 full texts, as well as other checks on unusual results, e.g., extremely wide confidence intervals. The complete codes and data are available at https://github.com/jdwren/ASEC and https://github.com/agbarnett/intervals.

We excluded the 0.1% of lower intervals that were zero, assuming this was an error by the authors. We excluded the 0.7% of abstract intervals and 0.9% of full-text intervals where the mean was not within the confidence interval, as this meant either the interval or mean (or both) was incorrect.

We used a second set of published estimates from health research based on thousands of analyses with no p-hacking or publication bias [13]. Using four large databases of insurance claims, the study examined all possible pairs of 17 treatments for depression using 22 outcomes. Risks were estimated using survival analysis, giving hazard ratios. Treatment–Outcome pairs were categorised as "negative" where there was no evidence in the literature of any association. "Positive" associations were created by simulating an increased risk with a hazard ratio of 1.5. Details are in the paper by Schuemie et al [13], the key point for our analysis is that we have a large sample of ratio confidence intervals from Treatment–Outcome pairs that are not subject to any "significance seeking" by researchers or journals.

Statistical methods

To summarise the intervals we plotted the cumulative empirical distribution of the lower and upper confidence interval limits. We used the cumulative distribution because it highlights a change in the distribution, which is our key interest, and it does not require any user-defined tuning parameters. Histograms require the selection of a binwidth and density plots require user-defined smoothing parameters, which are subjective and will create different impressions depending on the choice made. We include histograms in the online supplement using a binwidth of 0.1, but our results focus on the cumulative distributions.

Our hypothesis was that there would be a large change in the intervals near 1, which is the

For peer review only - http://bmjopen.bmj.com/site/about/guidelines.xhtml

commonly used null hypothesis of no difference on a ratio scale. A lower confidence interval limit for a ratio estimate that is greater than 1 would mean a statistically significant result, as would an upper interval limit below 1. To look for changes over time, we plotted separate cumulative densities in five year periods.

We used R version 3.6.0 for all analyses [14].

Patient and Public Involvement

No patients or members of the public were involved in this study.

Results

Our sample had over 968,000 intervals extracted from *Medline* abstracts from over 5,900 journals, and over 350,000 intervals extracted from the full-text from over 2,700 journals. For 11% of intervals the level of the confidence interval could not be determined, most often because it was not given. Where an interval could be determined, 99.7% were 95% confidence intervals, which corresponds to the commonly used p-value threshold of 0.05.

We found a clear and sudden change in the cumulative distribution of both lower and upper interval limits at the statistically significant threshold of 1 (Figure 1). There was a steep increase in the number of lower interval limits just above 1, meaning they were just above the threshold for statistical significance. Similarly there was a steep increase in the number of upper interval limits just below 1, and so just inside the statistically significant threshold. The discontinuity at 1 appears slightly stronger (less smooth) in the abstract than the full-text and there are noticeably more lower intervals below 1 in the full-text, which suggests that the biases are stronger in the abstract than the full-text.

If there was no p-hacking and publication bias, the cumulative distributions would be smooth S-shapes with no discontinuities, and this is confirmed in Figure 2 using the data without these biases.

The bias for statistically significant results in abstracts has persisted over time as shown by the five-year plots in Figure 3. The plots also show the reduced use of rounded intervals over time, as the cumulative densities are smoother in later years.

The online supplement contains sensitivity analyses that: use wider and narrow ratio axes; show histograms instead of cumulative densities; and show plots of the cumulative densities over time for the full-text. All the plots show the same striking changes in the number of intervals near 1.

For peer review only - http://bmjopen.bmj.com/site/about/guidelines.xhtml

Discussion

The strong bias for statistically significant results is clearly visible in the confidence intervals of a large sample of abstracts and full-texts from health and medical journals and has persisted for decades. This bias undermines the evidence base and impairs good decision making by giving a false picture of the effectiveness of treatments and procedures. Such biased evidence can harm patients and the public when it gets translated into policy and practice.

Confidence intervals have been promoted as a way of avoiding the simplistic bright-line thinking that often accompanies p-values. However, our results show that even though people are frequently reporting confidence intervals, many are still focusing on statistical significance. A push towards the greater use of confidence intervals in journals in place of p-values may therefore not solve the ongoing problem of bright-line thinking [15].

Large improvements in research practice are needed to improve the quality of evidence. Study results should be presented and published regardless of their statistical significance. This could be achieved by a wider uptake of study pre-registration and analysis plans [16], which reduces the potential for researchers to change their hypothesis or primary outcome after seeing the data.

Statistical significance should no longer be used as a tool to screen what results are published and the evidence base would be in a better state if significance were given far less prominence [17]. The prominence given to statistical significance is ironic given the widespread misunderstandings of its true meaning [2, 12].

Limitations

We did not aim to develop a new statistical test for p-hacking or numerically compare the extent of p-hacking in: abstracts versus full-texts, or our sample of the published literature versus the sample with no p-hacking. Instead our aim was to present some simple plots that highlight the extent of the problem.

We included all confidence intervals, but we anticipate the biases would be even more striking if we were able to restrict the sample to the primary outcomes of interest [18]. This is because the statistical significance of the primary outcomes dictate the “success” or “failure” of the study, and less high-profile comparisons (e.g., between groups at baseline) are less likely to be p-hacked.

For peer review only - http://bmjopen.bmj.com/site/about/guidelines.xhtml

We have taken a broad look at the combined evidence and have not examined individual studies to show that they were biased, nor did we estimate the size of the bias. Instead, we aimed to show the extent of the bias across the literature using a simple graphical method. To our knowledge this is the first paper to examine the problem using confidence intervals and uses one of the largest sample sizes including results from both abstracts and full-texts. For us the graphs are a warning of the urgent need for action to improve research practices.

Footnotes

Funding statement: AGB was supported by the National Health and Medical Research Council grant number APP1117784.

Competing interests statement: None declared.

Data sharing statement: The dataset and code are freely available on *github* at https://github.com/jdwren/ASEC and https://github.com/agbarnett/intervals.

Contributors: AB and JW conceived the idea. JW wrote the programs to extract the data; AB wrote the analyses code. AB wrote the first draft of the paper with comments from JW. AB is responsible for the overall content as is the guarantor.

Figure legends

Figure 1: Empirical cumulative distributions for ratio confidence intervals from *Medline* abstracts and full papers. To be statistically significant, lower intervals need to be above 1 and upper intervals need to be under 1 (vertical lines). The x-axes are restricted to ratios between 0.25 and 4.

Figure 2: Empirical cumulative distributions for ratio confidence interval limits from a sample with no p-hacking or publication bias. "Negative" results were for associations with no known evidence of an association and "positive" studies were simulated using an increased risk. The x-axes are restricted to ratios between 0.25 and 4.

Figure 3: Empirical cumulative distributions for ratio confidence intervals from *Medline* abstracts in five year intervals. The x-axes are restricted to ratios between 0.25 and 4.

References

1. Gwendolyn B. Emerson, Winston J. Warme, Fredric M. Wolf, James D. Heckman, Richard A. Brand, and Seth S. Leopold. Testing for the presence of positive-outcome bias in peer review. *Archives of Internal Medicine*, 170(21), 2010.

For peer review only

For peer review only - http://bmjopen.bmj.com/site/about/guidelines.xhtml

2. S. Ziliak and D.N. McCloskey. *The Cult of Statistical Significance: How the Standard Error Costs Us Jobs, Justice, and Lives*. University of Michigan Press, 2008.

3. Uri Simonsohn, Leif D. Nelson, and Joseph P. Simmons. p-curve and effect size. *Perspectives on Psychological Science*, 9(6):666–681, 2014.

4. Megan L. Head, Luke Holman, Rob Lanfear, Andrew T. Kahn, and Michael D. Jennions. The extent and consequences of p-hacking in science. *PLOS Biology*, 13(3):1–15, 03 2015.

5. Andrew Gelman and Eric Loken. The garden of forking paths: Why multiple comparisons can be a problem, even when there is no "fishing expedition" or "p-hacking" and the research hypothesis was posited ahead of time. http://www.stat.columbia.edu/~gelman/research/unpublished/p_hacking.pdf 2013.

6. Aled M. Edwards, Ruth Isserlin, Gary D. Bader, Stephen V. Frye, Timothy M. Willson, and Frank H. Yu. Too many roads not taken. *Nature*, 470(7333):163–165, 2011.

7. David Chavalarias, Joshua David D. Wallach, Alvin Ho Ting H. Li, and John P. Ioannidis. Evolution of reporting P values in the biomedical literature, 1990–2015. *JAMA*, 315(11):1141–1148, 2016.

8. Constantin Georgescu and Jonathan D Wren. Algorithmic identification of discrepancies between published ratios and their reported confidence intervals and p-values. *Bioinformatics*, 34(10):1758–1766, 2017.

9. J. Ranstam. Why the p-value culture is bad and confidence intervals a better alternative. *Osteoarthritis and Cartilage*, 20(8):805–808, 2012.

10. Ronald L. Wasserstein and Nicole A. Lazar. The ASA's statement on p-values: Context, process, and purpose. *The American Statistician*, 70(2):129–133, 2016.

11. Dong Kyu Lee. Alternatives to p-value: confidence interval and effect size. *Korean Journal of Anesthesiology*, 69(6):555, 2016.

12. Steven Goodman. A dirty dozen: Twelve p-value misconceptions. *Seminars in Hematology*, 45(3):135–140, 2008.

13. Martijn J. Schuemie, Patrick B. Ryan, George Hripcsak, David Madigan, and Marc A. Suchard. Improving reproducibility by using high-throughput observational studies with empirical calibration. *Philosophical Transactions of the Royal Society A: Mathematical, Physical and Engineering Sciences*, 376(2128):20170356, 2018.

14. R Core Team. *R: A Language and Environment for Statistical Computing*. R Foundation for Statistical Computing, Vienna, Austria, 2018.

15. Fiona Fidler, Neil Thomason, Geoff Cumming, Sue Finch, and Joanna Leeman. Editors

For peer review only

can lead researchers to confidence intervals, but can't make them think. *Psychological Science*, 15(2):119–126, 2004.

16. Robert M. Kaplan and Veronica L. Irvin. Likelihood of null effects of large NHLBI clinical trials has increased over time. *PLOS ONE*, 10(8):e0132382, 2015.

17. Blakeley B. McShane and David Gal. Statistical significance and the dichotomization of evidence. *Journal of the American Statistical Association*, 112(519):885–895, 2017.

18. Uri Simonsohn. [41] falsely reassuring: Analyses of ALL p-values, http://datacolada.org/41 2015.

For peer review only

For peer review only - http://bmjopen.bmj.com/site/about/guidelines.xhtml

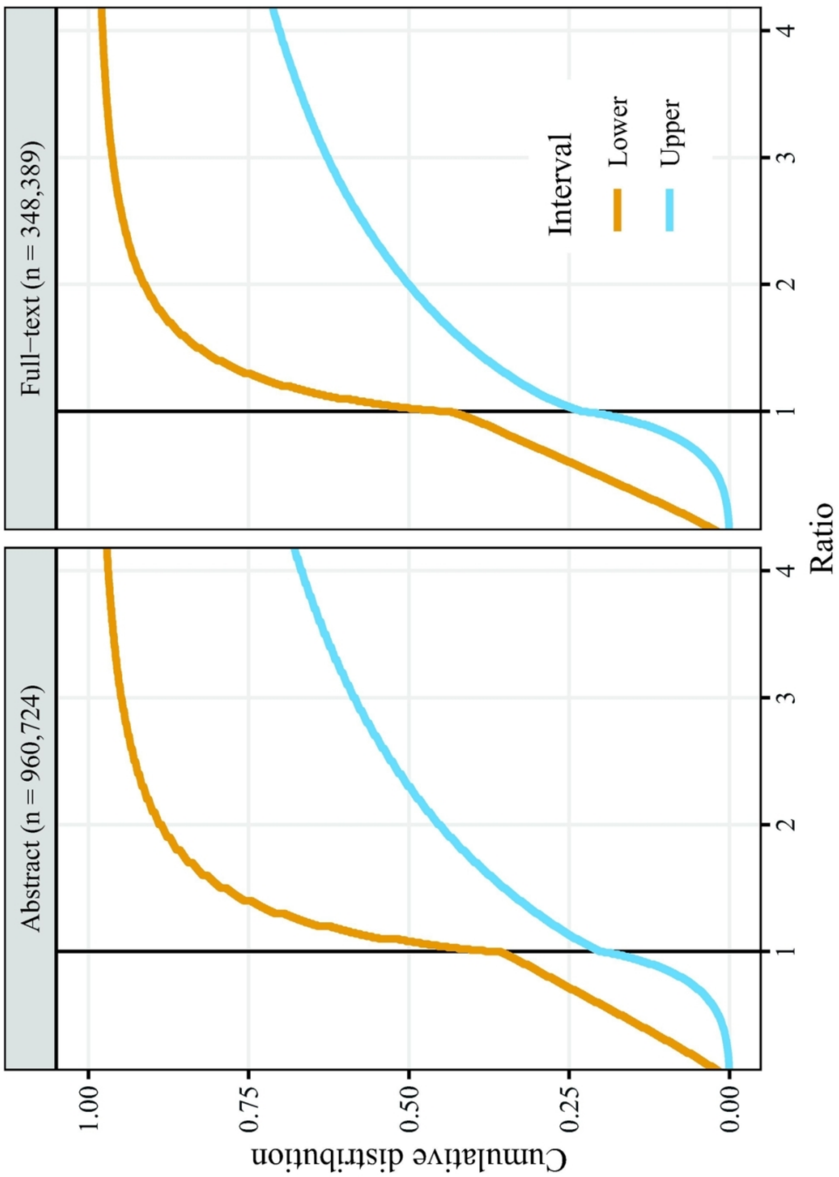

Empirical cumulative distributions for ratio confidence intervals from Medline abstracts and full papers. To be statistically significant, lower intervals need to be above 1 and upper intervals need to be under 1 (vertical lines). The x-axes are restricted to ratios between 0.25 and 4.

101x140mm (300 x 300 DPI)

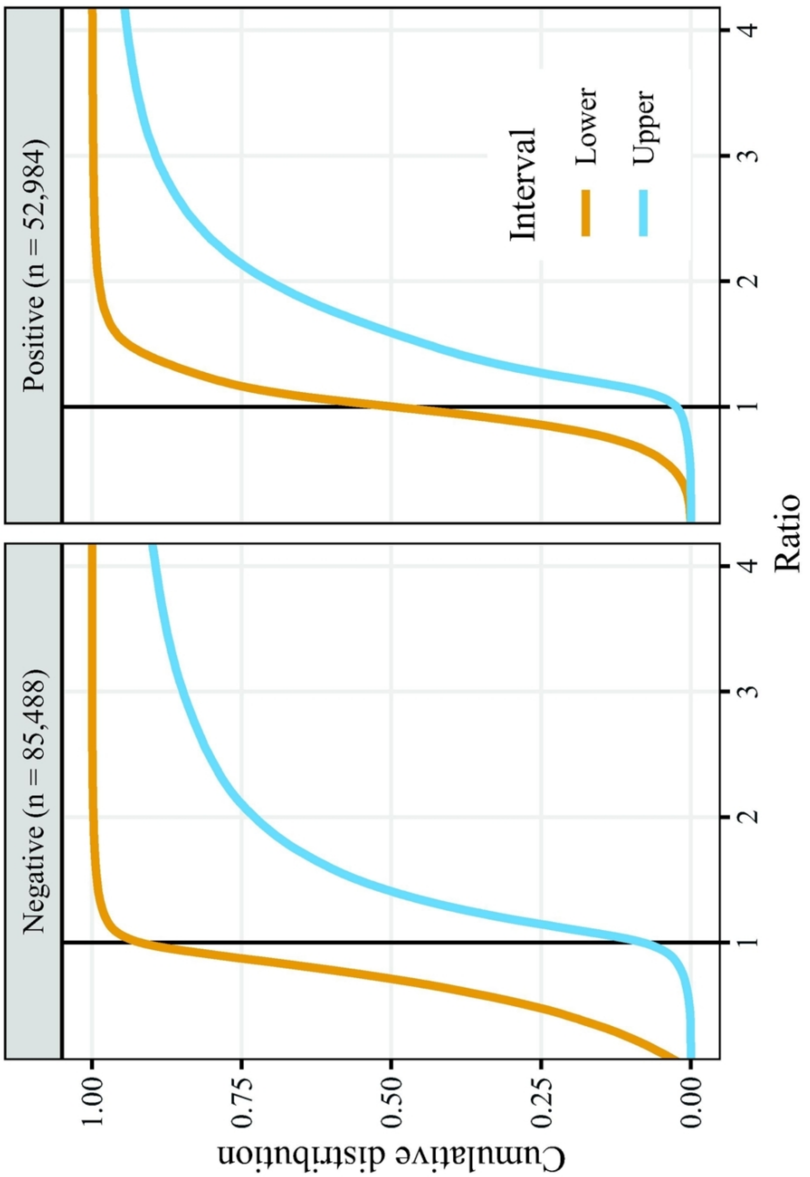

Empirical cumulative distributions for ratio confidence interval limits from a sample with no p-hacking or publication bias. "Negative" results were for associations with no known evidence of an association and "positive" studies were simulated using an increased risk. The x-axes are restricted to ratios between 0.25 and 4.

88x127mm (300 x 300 DPI)

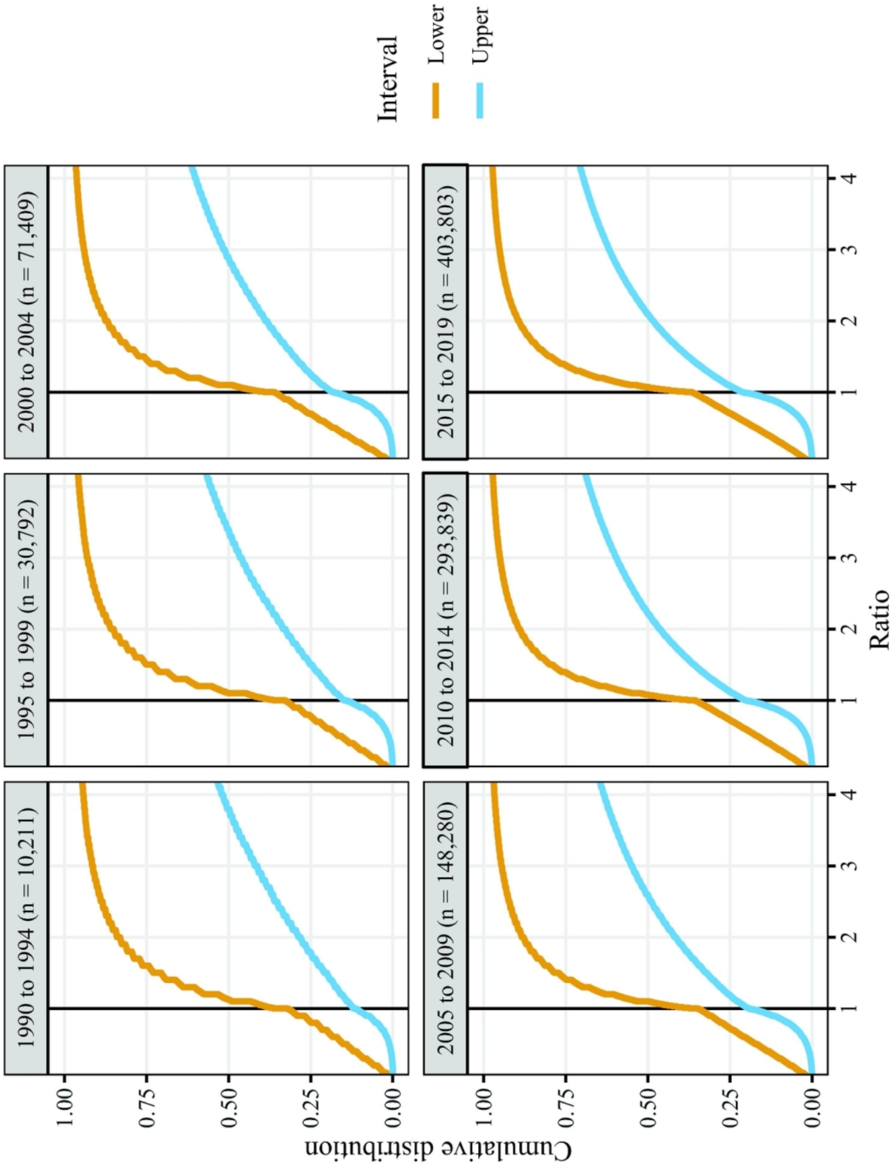

Empirical cumulative distributions for ratio confidence intervals from Medline abstracts in five year intervals. The x-axes are restricted to ratios between 0.25 and 4.

127x165mm (300 x 300 DPI)

Confidence intervals in health and medical journals show an implausible excess of statistically significant results

Adrian Barnett and Jonathan Wren

S1. Histograms

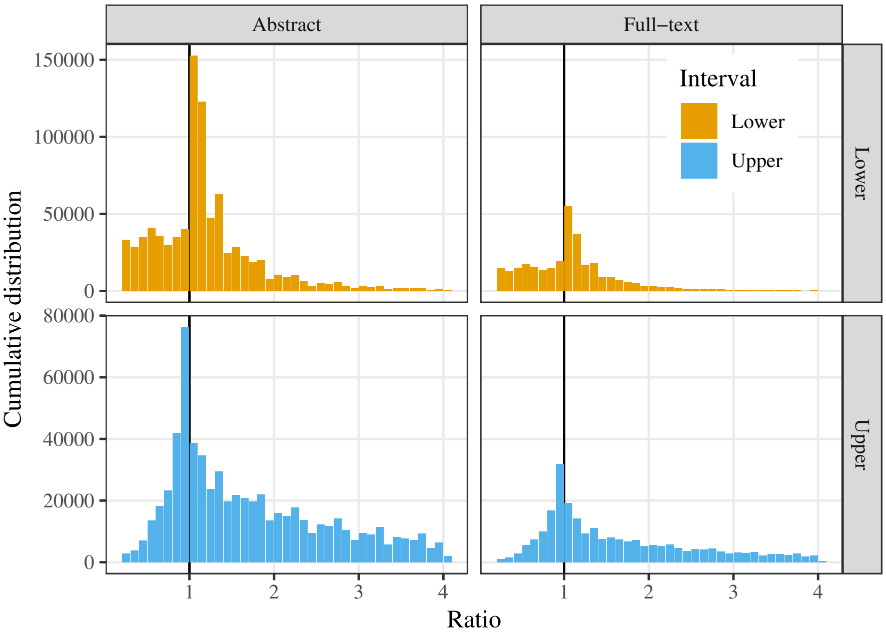

Figure S.1: Histograms for ratio confidence intervals from *Medline* abstracts and full-texts. The ratios are limited to 0.2 to 4.0.

The results in the figure above are an alternative version of the main results using a histogram rather than a cumulative density plot. We used binwidths of 0.1. The plot shows the dramatic rise in intervals just above 1 for the lower intervals, and rise in intervals below 1 for the upper intervals. The downside to using histograms is the requirement to select the binwidth and the plot's appearance does depend on this choice.

For peer review only - http://bmjopen.bmj.com/site/about/guidelines.xhtml

S2. Plots using a log-scale

In the results we focused on the key intervals close to 1 by restricting the intervals to between 0.25 and 4, which had the consequence of excluding 11% of lower intervals and 33% of upper intervals. The plot below uses a log-scale (base e) for the intervals which allows us to show ratios from 0.1 to 10. This plot excludes 4% of lower intervals and 15% of upper intervals.

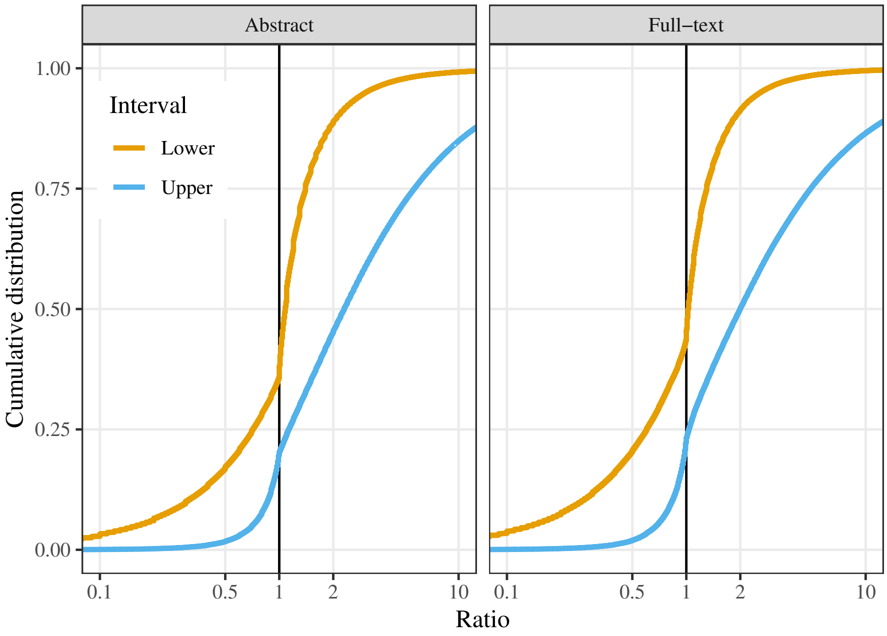

Figure S.2: Empirical cumulative distributions for ratio confidence intervals from *Medline* abstracts and full-texts. To be statistically significant, lower intervals need to be above 1 and upper intervals need to be under 1 (vertical lines). The x-axes are on a log-scale (base e).

The figure shows the same dramatic change close to 1 for the lower and upper intervals. One advantage of showing more of the data is that it confirms that the only discontinuity is at a ratio of 1.

For peer review only - http://bmjopen.bmj.com/site/about/guidelines.xhtml

S3. Intervals in a narrow range close to 1

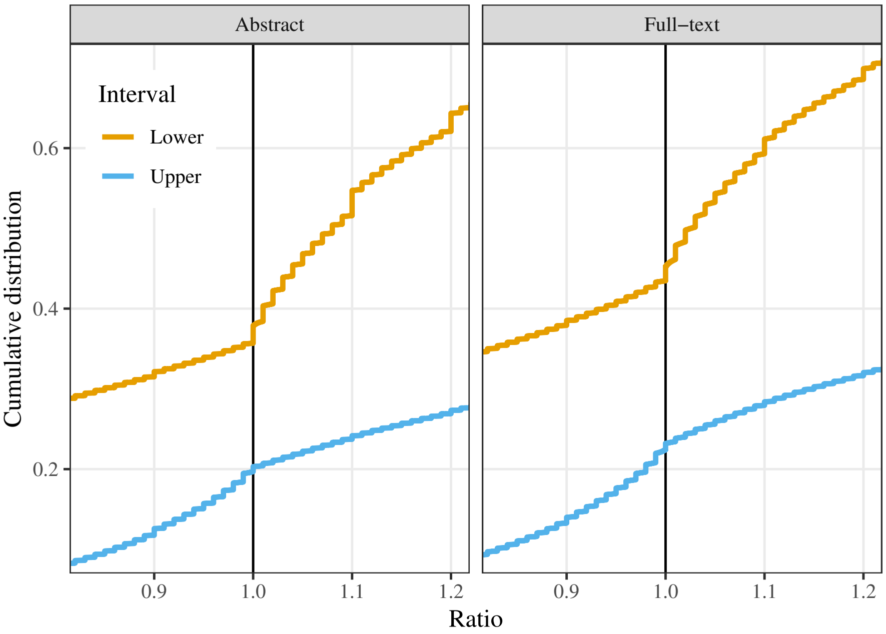

Figure S.3: Empirical cumulative distributions for ratio confidence intervals from *Medline* abstracts and full-texts. The ratios are limited to the narrow range of 0.83 to 1.2. The y-axes do not start at zero.

The plot above is an alternative look at the main results which examines those intervals close to 1. The x-axes are restricted to 0.83 to 1.2 and the y-axis are restricted to reduce white space. The plot includes 35% of all lower intervals and 20% of all upper intervals. The steps in the ratios are due to results being presented to 1 decimal place. The discontinuity in the cumulative numbers at 1 is still clearly visible and hence the changes shown in previous figures are not simply due to rounding.

For peer review only - http://bmjopen.bmj.com/site/about/guidelines.xhtml

S4. Full-text results over time

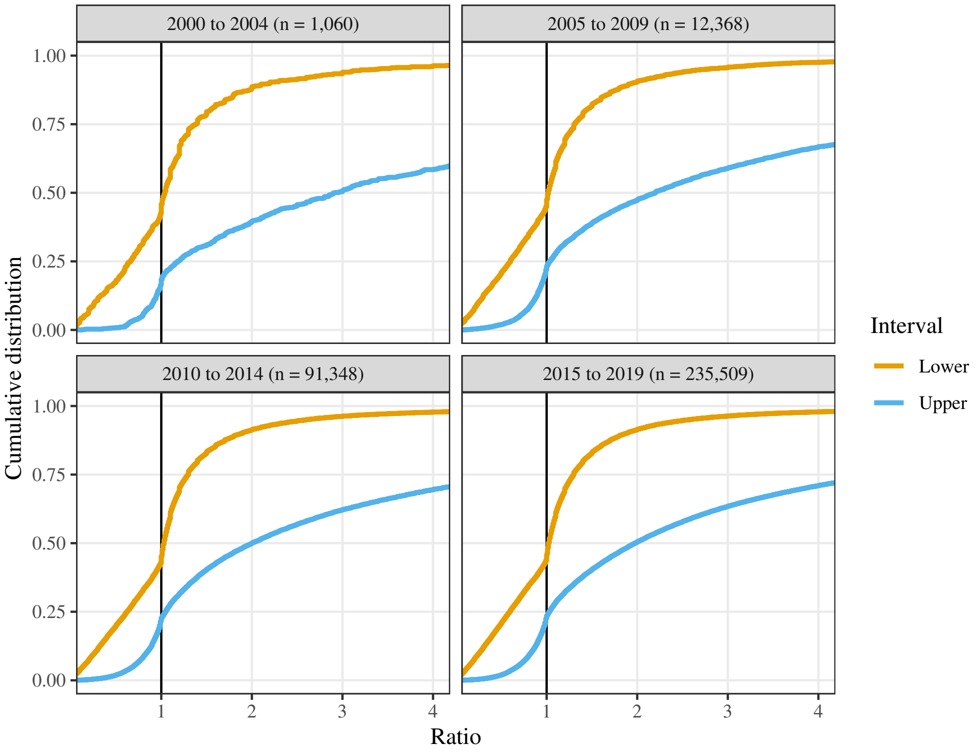

Figure S.4: Empirical cumulative distributions for ratio confidence intervals from full-texts in five year intervals. To be statistically significant, lower intervals need to be above 1 and upper intervals need to be under 1 (vertical lines).

The figure above is the complement to Figure 3 in the main paper, which examined the results over time from abstracts. This plot has a narrower time range because reasonable numbers of confidence intervals could only be extracted from the year 2000 onward. There was little change over time in the cumulative distributions, indicating little improvement in the bias for statistically significant results.

For peer review only - http://bmjopen.bmj.com/site/about/guidelines.xhtml

Reporting checklist for cross sectional study.

Based on the STROBE cross sectional guidelines.

Instructions to authors

Complete this checklist by entering the page numbers from your manuscript where readers will find each of the items listed below.

Your article may not currently address all the items on the checklist. Please modify your text to include the missing information. If you are certain that an item does not apply, please write "n/a" and provide a short explanation.

Upload your completed checklist as an extra file when you submit to a journal.

In your methods section, say that you used the STROBE cross sectional reporting guidelines, and cite them as:

von Elm E, Altman DG, Egger M, Pocock SJ, Gotzsche PC, Vandenbroucke JP. The Strengthening the Reporting of Observational Studies in Epidemiology (STROBE) Statement: guidelines for reporting observational studies.

		Reporting Item	Page Number
Title	#1a	Indicate the study's design with a commonly used term in the title or the abstract	1
Abstract	#1b	Provide in the abstract an informative and balanced summary of what was done and what was found	2
Background / rationale	#2	Explain the scientific background and rationale for the investigation being reported	2-3
Objectives	#3	State specific objectives, including any prespecified hypotheses	3
Study design	#4	Present key elements of study design early in the paper	3-4
Setting	#5	Describe the setting, locations, and relevant dates, including periods of recruitment, exposure, follow-up, and data collection	4
Eligibility criteria	#6a	Give the eligibility criteria, and the sources and methods of selection of participants.	4

For peer review only

	#7	Clearly define all outcomes, exposures, predictors, potential confounders, and effect modifiers. Give diagnostic criteria, if applicable	4-5
Data sources / measurement	#8	For each variable of interest give sources of data and details of methods of assessment (measurement). Describe comparability of assessment methods if there is more than one group. Give information separately for for exposed and unexposed groups if applicable.	4-5
Bias	#9	Describe any efforts to address potential sources of bias	n/a
Study size	#10	Explain how the study size was arrived at	See note 1
Quantitative variables	#11	Explain how quantitative variables were handled in the analyses. If applicable, describe which groupings were chosen, and why	4-5
Statistical methods	#12a	Describe all statistical methods, including those used to control for confounding	4-5
	#12b	Describe any methods used to examine subgroups and interactions	See note 2
	#12c	Explain how missing data were addressed	See note 3
	#12d	If applicable, describe analytical methods taking account of sampling strategy	See note 4
	#12e	Describe any sensitivity analyses	Supplementary file
Participants	#13a	Report numbers of individuals at each stage of study—eg numbers potentially eligible, examined for eligibility, confirmed eligible, included in the study, completing follow-up, and analysed. Give information separately for for exposed and unexposed groups if applicable.	5
	#13b	Give reasons for non-participation at each stage	See note 6
	#13c	Consider use of a flow diagram	See note 7
Descriptive data	#14a	Give characteristics of study participants (eg demographic, clinical, social) and information on exposures and potential confounders. Give information	See note 8

For peer review only

		separately for exposed and unexposed groups if applicable.	
	#14b	Indicate number of participants with missing data for each variable of interest	See note 9
Outcome data	#15	Report numbers of outcome events or summary measures. Give information separately for exposed and unexposed groups if applicable.	See note 10
Main results	#16a	Give unadjusted estimates and, if applicable, confounder-adjusted estimates and their precision (eg, 95% confidence interval). Make clear which confounders were adjusted for and why they were included	See note 11
	#16b	Report category boundaries when continuous variables were categorized	See note 12
	#16c	If relevant, consider translating estimates of relative risk into absolute risk for a meaningful time period	See note 13
Other analyses	#17	Report other analyses done—e.g., analyses of subgroups and interactions, and sensitivity analyses	See note 14
Key results	#18	Summarise key results with reference to study objectives	6
Limitations	#19	Discuss limitations of the study, taking into account sources of potential bias or imprecision. Discuss both direction and magnitude of any potential bias.	7
Interpretation	#20	Give a cautious overall interpretation considering objectives, limitations, multiplicity of analyses, results from similar studies, and other relevant evidence.	7
Generalisability	#21	Discuss the generalisability (external validity) of the study results	6
Funding	#22	Give the source of funding and the role of the funders for the present study and, if applicable, for the original study on which the present article is based	7

For peer review only

Author notes

1. n/a, used all available data from Medline

2. n/a, no subgroups or interactions

3. n/a, no missing data

4. n/a, was a census of all available data

5. n/a, no sensitivity analyses

6. n/a, no stages of participation

7. n/a, simple cross-sectional study with no stages

8. n/a, no real characteristics and there are there are no potential confounders or exposures

9. n/a, no missing data

10. n/a, no events

11. n/a, no statistical modelling

12. n/a, no boundaries used

13. n/a, no relative risk calculations

14. n/a, no additional analyses

The STROBE checklist is distributed under the terms of the Creative Commons Attribution License CC-BY. This checklist was completed on 28. February 2019 using https://www.goodreports.org/, a tool made by the EQUATOR Network in collaboration with Penelope.ai

For peer review only
